# Deciphering the Contribution of ROCK-Dependent Actin Cytoskeleton Remodeling to Testosterone Production in Mouse Leydig Cells

**DOI:** 10.3390/cells14231868

**Published:** 2025-11-26

**Authors:** Ke Xu, Fengze Sun, Yuwei Hu, Ning Hou, Shan Wang, Chengzi Huang

**Affiliations:** 1 Department of Reproductive Medicine, Shandong Provincial Hospital Affiliated to Shandong First Medical University, Jinan 250021, China; 202020745@mail.sdu.edu.cn; 2 Shandong Key Laboratory of Reproductive Research and Birth Defect Prevention, Shandong First Medical University, Jinan 250021, China; 3 Jinan Engineering Laboratory of Reproductive Diagnosis and Treatment Technology, Shandong Provincial Hospital Affiliated to Shandong First Medical University, Jinan 250021, China; 4 School of Clinical and Basic Medicine, Shandong First Medical University and Shandong Academy of Medical Sciences, Jinan 250117, China; sunfz1806@163.com; 5 School of Basic Medical Sciences, Shandong University, Jinan 250012, China; 202316286@mail.sdu.edu.cn; 6 Department of Pharmacy, Shandong Provincial Hospital Affiliated to Shandong First Medical University, Jinan 250012, China; hou_ning@sina.com; 7 Center for Reproductive Medicine, Department of Obstetrics and Gynecology, Qilu Hospital, Shandong University, Jinan 250012, China

**Keywords:** actin remodeling, testosterone biosynthesis, ROCK, hCG, cholesterol trafficking, SREBP2

## Abstract

Infertility affects about 17.5% of couples, with male factors accounting for approximately 50% of cases. Cytoskeletal remodeling is increasingly recognized as a critical component of male reproductive function, particularly in the regulation of testosterone synthesis by Leydig cells. However, the underlying molecular mechanisms remain poorly defined. Rho-associated coiled-coil-containing kinase (ROCK), a key cytoskeletal regulator, influences actin dynamics, impacting intracellular trafficking. In this study, we investigated the roles of ROCK1 and ROCK2 in Leydig cells using the TM3 cell model. Pharmacological inhibition of ROCK activity with Y-27632 impaired actin cytoskeleton organization, reduced the phosphorylation of LIMK, COFILIN, and MLC2, and disrupted the colocalization of F-actin with StAR and cholesterol, thereby decreasing testosterone production. Furthermore, RNA-seq revealed that hCG promotes transcription of steroidogenesis-related genes, while ROCK inhibition reverses this effect. Silencing of ROCK1 via siRNA mimicked the effects of ROCK-i, suppressing steroidogenic gene expression and testosterone synthesis. In contrast, ROCK2 knockdown enhanced testosterone secretion, promoted F-actin remodeling, and increased traffic of cholesterol targeting mitochondria. These opposing effects triggered distinct responses in the SCAP–SREBP2 axis, indicating a feedback mechanism regulating cholesterol homeostasis. Collectively, our findings uncover the isoform-specific roles of ROCK1 and ROCK2 in coordinating cytoskeletal dynamics and steroidogenic activity, providing new insights into the regulation of male reproductive endocrinology and identifying potential therapeutic targets for androgen deficiency and male infertility.

## 1. Introduction

The issue of infertility constitutes a significant challenge to male reproductive health worldwide, with approximately 17.5% of couples globally experiencing difficulties conceiving in their lifetimes [1]. Testosterone is the leading endocrine factor in orchestrating the intricacies of male reproductive physiology, governing pivotal processes such as spermatogenesis and the modulation of sexual function. Leydig cells, situated within the interstitial compartment amid the seminiferous tubules, emerge as central protagonists in the intricate cascade of androgen biosynthesis, serving as the principal site for the production of testosterone [2]. Upon gonadotropin-releasing hormone (GnRH) stimulation from the hypothalamus, pituitary luteinizing hormone (LH) or human chorionic gonadotropin (hCG) binds to Leydig cell luteinizing hormone/chorionic gonadotrophin receptor (LHCGR), activating G protein cascade signaling. This triggers cyclic adenosine 3′,5′-monophosphate (cAMP) production, facilitating cholesterol translocation from lipid droplets or plasma membrane into mitochondria—a pivotal and rate-limiting step in biosynthesis of steroid hormones [3]. Subsequently, cholesterol is converted to pregnenolone within mitochondria and metabolized into testosterone by enzymes located in the smooth endoplasmic reticulum (ER). Several molecular valves are implemented to modulate testosterone production in accordance with cellular demands, for instance, steroidogenic acute regulatory protein (StAR) governs the traffic of cholesterol towards the inner membrane of mitochondria [4]. The coordinated modulations in this process are intricately linked with another essential component, the cytoskeleton, which serves a critical role in facilitating intra-cellular traffic [5]. Notwithstanding some investigations have provided evidence for the involvement of the cytoskeleton in steroidogenesis, encompassing cholesterol delivery and mitochondrial positioning, much of the molecular basis underlying the modulation in this process, such as inter-organelle substrate trafficking, still need to be uncovered.

In the regulation network of cytoskeletal components, the Rho-associated protein kinase (ROCK), which consists of two isoforms namely ROCK1 and ROCK2 in mammals, emerges as a master orchestrator, governing not only the dynamic remodeling of the actin cytoskeleton organization but also exerting stringent control over vesicular transport processes [6]. As a downstream effector of Rho GTPase, ROCK is acknowledged for its role in regulating the reorganization and stabilization of actin filaments, primarily through the promotion of stress fibers assembly and formation of focal adhesions [7]. Through the phosphorylation of a divergent group of downstream targets, including LIM kinase (LIMK), myosin light chain (MLC), MAP2/Tau, and Doublecortin, etc. [8], activated ROCK proteins have been implicated in many biological processes such as cell migration, apoptosis and cytokinesis via a series of signaling cascades [9,10]. Notably, steroidogenesis in various cellular models has frequently been causally associated with actin cytoskeleton, as indicated by the inhibitory effects observed on steroidogenesis upon exposure to compounds that disrupt actin cytoskeletal reorganization [11,12]. In addition, the role of RhoA/ROCK1 as a actin cytoskeletal organizer in the regulation of mammalian ovarian steroidogenesis has recently been documented [13]. Interestingly, co-inhibition of phosphodiesterase 4 (PDE4) and phosphodiesterase 8 (PDE8), known stimulators of cAMP-dependent steroidogenesis, was observed to activate RhoA in MA-10 mouse Leydig cells. This activation was accompanied by significant phosphorylation events in Arhgap17 and Arhgef2, both of which are recognized regulators of Rho activity. Proteomic analysis revealed a plethora of Rho-GTPase signaling pathways significantly enriched in MA-10 cells [14]. Furthermore, in vivo inhibition of Rho function led to a reduction in hCG-induced testosterone production in mice [15]. These observations offer novel perspectives on the participation of Rho GTPases in testicular steroid hormone production. Nonetheless, the mechanism through which ROCK signaling contributes to testosterone production remains elusive, underscoring the necessity for the comprehension of the involvement of cytoskeletal substance transport in steroid hormone biosynthesis.

In the current investigation, we explored the role of ROCK-dependent actin remodeling in testosterone production by modulating ROCK activity using Y-27632, a selective ROCK inhibitor known for abolishing RhoA-induced formation of stress fibers and focal adhesions, in TM3 mouse Leydig cells. The TM3 cell line, derived from immature mouse Leydig cells [16], has been widely used to study testosterone biosynthesis due to its ability to respond to LH/hCG stimulation and secrete testosterone. Unlike MA-10 cells, which primarily produce progesterone due to the lack of CYP17A1 expression [3], TM3 cells retain the enzymatic machinery necessary for testosterone production. Although their hormone production capacity is lower than that of primary Leydig cells, TM3 cells remain a convenient and reliable in vitro model for dissecting steroidogenic signaling pathways in mouse Leydig cells. By evaluating the phosphorylation status of canonical ROCK substrates (LIMK, COFILIN, and MLC2) via immunoblotting and assessing cytoskeletal organization through immunofluorescence, we demonstrated that ROCK inhibition disrupted actin remodeling and altered the subcellular localization of key steroidogenic enzymes, such as 3β-HSD and StAR. In hCG-treated cells, increased ROCK1 phosphorylation was accompanied by upregulation of several enzymes essential for testosterone biosynthesis—effects that were significantly attenuated by ROCK inhibition. Moreover, cholesterol trafficking to mitochondria and the colocalization of StAR with F-actin were impaired under ROCK inhibition, leading to reduced testosterone secretion. To dissect isoform-specific functions, we employed siRNA-mediated knockdown of ROCK1 and ROCK2. Notably, ROCK1 silencing reduced testosterone production, disrupted cytoskeletal integrity, and suppressed steroidogenic gene expression. In contrast, ROCK2 knockdown enhanced testosterone synthesis, promoted cortical F-actin accumulation, increased mitochondrial cholesterol targeting, and upregulated the SCAP–SREBP2 axis. These opposing effects indicate that ROCK1 and ROCK2 differentially regulate cholesterol transport and homeostasis, likely through distinct feedback mechanisms involving cytoskeletal remodeling and lipid metabolism. Together, these findings suggest that hCG-LHCGR signaling activates the Rho/ROCK pathway, which in turn modulates actin cytoskeletal dynamics and coordinates the expression, localization, and function of steroidogenic factors. This study reveals the divergent roles of ROCK isoforms in testosterone biosynthesis and highlights their potential as therapeutic targets for male reproductive disorders.

## 2. Materials and Methods

### 2.1. Cell Culturing and Stimulation

The mouse Leydig cell line TM3 (National Collection of Authenticated Cell Cultures) were grown in Dulbecco’s Modified Eagle Medium (DMEM, Gibco, ThermoFisher Scientific, Grand Island, NY, USA) containing 5% fetal bovine serum (FBS, Gibco, ThermoFisher Scientific, Grand Island, NY, USA) and 1% penicillin–streptomycin (Invitrogen, ThermoFisher Scientific, Waltham, MA, USA) at 37 °C with 5% CO_2_. Adherent cells were completely refreshed by aspirating old medium, washing 2–3 times with sterilized PBS solution (Gibco, ThermoFisher Scientific, Grand Island, NY, USA), and adding fresh medium. For passaging, cells were washed with PBS, treated with trypsin (Gibco, Thermo Fisher Scientific, Grand Island, NY, USA) until rounding was observed under a microscope, quenched with an equal volume of medium, and gently resuspended. The cell suspension was then centrifuged at 1000× *g* for 5 min, supernatant discarded, and cells resuspended in fresh medium for seeding into new culture dishes. For cryopreservation, cells were washed with PBS, digested with trypsin, quenched, resuspended, and centrifuged at 1000× *g*. The pellet was resuspended in serum-free cryopreservation solution (New Cell & Molecular Biotech, Suzhou, China) and aliquoted into cryovials for storage in liquid nitrogen. To assess the testosterone production capacity of TM3 cells, 1 × 10^5^ cells per well were seeded in a 12-well plate. The ability of these cells to produce testosterone was then evaluated following stimulation with 10 IU/mL human chorionic gonadotropin (hCG, Sigma-Aldrich, Darmstadt, Germany) for 6 or 12 h, a concentration consistent with previous studies in comparable in vitro models demonstrating effective stimulation of steroidogenic pathways [17,18], using DMEM supplemented with 5% FBS. To pharmacologically inhibit ROCK, the compound Y-27632 (MedChemExpress, Monmouth Junction, NJ, USA) was employed. Initially, Y-27632 was dissolved in dimethyl sulfoxide (DMSO) to prepare stock solutions, which were then further diluted in cell culture media to achieve the final concentration of 10 μM for experimental use. Mouse TM3 Leydig cells were treated with Y-27632 to evaluate its effects. To test the impact of Y-27632 on testosterone levels over time, samples were collected after hCG stimulation and subsequently treated with the ROCK inhibitor for 2 h or 4 h periods. To examine testosterone levels, cell culture medium was collected and stored at −20 °C. To assess the effects of the ROCK inhibitor on the cells, the drug was treated, with a control group received with an equivalent volume of DMSO as a negative control. Immediately afterward, the cells were either prepared for protein/RNA extraction or fixed for fluorescence staining.

### 2.2. Animals

The animals in this study were kept under controlled environmental conditions, with 12 h light cycles and unrestricted access to pathogen-free water and food. All breeding activities were conducted at the Model Animal Research Center of Shandong University, where the mouse colony was maintained, and samples were collected following protocols approved by the Animal Ethics Committee of the School of Medicine, Shandong University. Additionally, all animal care protocols in this study were reviewed and approved by the Animal Use Committee of the School of Medicine, Shandong University.

### 2.3. Antibodies and Reagents

The primary antibodies for Western Blot (WB) and immunofluorescent staining (IF) used in the study are listed in Table S1. HRP-conjugated Affinipure goat anti-mouse and anti-rabbit IgG (H + L) (SA00001-1/SA00001-2, Proteintech, Rosemont, IL, USA) was used as the sec-ondary antibodies for WB. The secondary antibodies used for immunofluorescent staining were Alexa Fluor 488/594/647-conjugated goat anti-rabbit IgG (H + L) (A-11008/A-11012/A-21244, Invitrogen, Waltham, MA, USA), Alexa Fluor 647-conjugated goat anti-mouse IgG (H + L) (A-21235, Invitrogen, Waltham, MA, USA), and goat anti-mouse IgG H&L (Alexa Fluor 488/594) (ab150117/ab150120, Abcam, Cambridge, UK). Rhodamine phalloidin was used to visualize F-actin (R415, Invitrogen, Waltham, MA, USA), MitoTracker Deep Red FM was used to label mitochondria (M22426, Invitrogen, Waltham, MA, USA), CholEsteryl BODIPY FL C12 was used to trace intracellular cholesterol (C3927MP, Invitrogen, Waltham, MA, USA) and Mounting Medium with DAPI was used for nuclear staining and coverslip mounting (ab104139, Abcam, Cambridge, UK).

### 2.4. siRNA-Mediated Knockdown of ROCK1 and ROCK2 in TM3 Cells

To investigate the isoform-specific roles of ROCK1 and ROCK2 in Leydig cell function, small interfering RNAs (siRNAs) targeting mouse *Rock1* and *Rock2* (GenePharma, Shanghai, China) were used to selectively silence each gene in TM3 cells. A non-targeting siRNA served as the negative control (SiCtrl). Cells were seeded in 6-well or 12-well plates and transfected at ~60–70% confluence using Lipofectamine RNAiMAX (Invitrogen, Thermo Fisher Scientific, Waltham, MA, USA) according to the manufacturer’s protocol. Briefly, siRNA-lipid complexes were prepared in Opti-MEM (Gibco, ThermoFisher Scientific, Grand Island, NY, USA) and added to the culture medium to achieve a final siRNA concentration of 50 nM. After 24 h of transfection, cells were either harvested for RNA/protein extraction or subjected to subsequent hCG (10 IU/mL) stimulation for an additional 12 h before analysis. Knockdown efficiency was validated by qPCR and Western Blot.

### 2.5. Tissue Collection and Immunofluorescence

For histological and immunofluorescence analysis, tissue collection, dissection, fixation, dehydration, and embedding were conducted on at least three adult WT C57BL/6J mice as previously described [19]. Sections of 5 μm thickness were cut using a microtome (HistoCore BIOCUT, Leica Biosystems, Nussloch, Germany) and mounted onto glass slides. After drying, the sections were deparaffinized in xylene, rehydrated through a graded alcohol series, and immersed in sodium citrate buffer (pH 6.0). Antigen retrieval was performed by heating the sections in a boiling water bath for 30 min. Following permeabilization with PBS containing 0.3% Triton X-100 and blocking with 5% bovine serum albumin, the slides were incubated with primary antibodies overnight at 4 °C. The sections were then rinsed in PBS and incubated with FITC-conjugated secondary antibodies for 60 min at room temperature. Finally, the nuclei were visualized using a Mounting Medium with DAPI.

### 2.6. Immunocytochemistry (ICC) Fluorescence Staining

Cells were digested and seeded onto 12-well plates containing 0.18 mm coverslips. When cell confluence reached 50–70%, the medium was aspirated, and cells were washed twice with cold PBS, followed by fixation with 4% PFA at room temperature for 20 min. The fixed coverslips were washed with PBS. Permeabilization was carried out using PBS containing 0.3% Triton X-100 for 40 min, followed by washing with PBS. Blocking was performed with immunofluorescence blocking buffer for 40 min to 1 h. After aspirating the blocking buffer, the primary antibody, diluted in immunofluorescence primary antibody dilution buffer, was added and incubated overnight at 4 °C in a humidified chamber. The cells were then washed with PBS. Subsequently, the secondary antibody, diluted in PBS, was added and incubated at 37 °C in the dark for 1 h. Following another series of PBS washes, the coverslips were mounted onto glass slides with Mounting Medium with DAPI for nuclear counterstaining. The slides were sealed with nail polish and examined under a fluorescence microscope.

### 2.7. Protein Extraction and Western Blot Analysis

Harvested cells or testis tissues from adult mice were transferred into pre-cooled denaturing buffer from the Minute Total Protein Extraction Kit (SD-001, Invent Biotech, Plymouth, MN, USA), containing protease inhibitors (CO-RO, Roche, Indianapolis, IN, USA) and phosphatase inhibitors (PHOSS-RO, Roche, Indianapolis, IN, USA). Protein extraction was performed following the manufacturer’s instructions. The protein concentration in the supernatant was measured using the Pierce BCA Protein Assay Kit (23225, ThermoFisher Scientific, Waltham, MA, USA) to ensure equal loading. After dilution with loading buffer and boiling for 10 min at 98 °C, protein lysate per sample was resolved by sodium dodecyl sulfate-polyacrylamide gel electrophoresis (SDS-PAGE). The electrophoresis was performed at 100 V constant voltage for 2 h and the separated proteins were transferred to polyvinylidene fluoride (PVDF) membranes, which were then blocked with noise-canceling reagents (WBAVDCH01, Millipore, Burlington, MA, USA). Blocking was proceeded at room temperature for 1–2 h or overnight at 4 °C. Membranes were incubated overnight at 4 °C with primary antibodies, followed by incubation with HRP-conjugated secondary antibodies. Immunodetection was achieved using a chemiluminescence imaging system (ChemiDoc MP, Bio-Rad Laboratories, Hercules, CA, USA).

### 2.8. RNA Extraction and Reverse Transcription-Quantitative Polymerase Chain Reaction (RT-qPCR)

Total RNA was extracted from adherent cells using the SteadyPure Quick RNA Extraction Kit (AG21023, Accurate Biotechnology, Changsha, China) following the manufacturer’s protocol. Cells were washed with PBS, lysed with 500 μL Buffer QLS, and detached using a cell scraper. The lysate was pipetted until clear, left at room temperature for 2 min, then mixed with an equal volume of ethanol and transferred to a Quick RNA Mini Column. After centrifugation at 12,000 rpm for 2 min, the column was washed with 700 μL Buffer QWB, centrifuged again, and transferred to a new tube. RNA was eluted with 30–200 μL RNase-free water and stored at −80 °C. RNA concentration and purity were measured using a NanoDrop spectrophotometer (840-317400, ThermoFisher Scientific, Waltham, MA, USA), and samples were either stored at −80 °C or used for reverse transcription. For the two-step reverse transcription and qPCR experiments, the Evo M-MLV RT Premix Kit (AG11728, Accurate Biotechnology, Changsha, China) and SYBR Green Pro Taq HS Premix qPCR Kit (AG11701, Accurate Biotechnology, Changsha, China) were used according to the manufacturer’s protocol. All reagents were RNase-free. For reverse transcription, 10 μL of the DNA removal mixture, 4 μL of 5× EVO M-MLV RT Reaction Mix Ver.2, and 6 μL of RNase-free water were used, with conditions set to 37 °C for 15 min, 85 °C for 5 s, and 4 °C hold. For qRT-PCR, the reaction mixture included 10 μL of 2X SYBR Green Pro Taq HS Premix, 0.4 μL each of 10 μM forward and reverse primers, cDNA template, and RNase-free water up to 20 μL. qPCR conditions were 95 °C for 30 s, 40 cycles of 95 °C for 5 s and 60 °C for 30 s, with a melting curve at 95 °C for 5 s, 65 °C for 1 min, and hold at 4 °C. Relative gene expression levels were calculated using the 2^−ΔΔCT^ method. The sequences for the qPCR primers used in the study are listed in Table S1.

### 2.9. Testosterone Detection by Enzyme-Linked Immunosorbent Assay (ELISA)

For the detection of testosterone in cell culture supernatants, the Testosterone ELISA Kit (582701, Cayman Chemical, Ann Arbor, MI, USA) was used, following the manufacturer’s protocol. Reagents such as ELISA buffer and wash buffer were prepared using ultrapure water (10977035, ThermoFisher Scientific, Waltham, MA, USA). After the preparation of testosterone standard, testosterone acetylcholinesterase tracer and testosterone ELISA antiserum working solutions were prepared. Following the assay, absorbance was read at 405–420 nm using a microplate reader (INFINITE 200 PRO, Tecan Group, Männedorf, Switzerland).

### 2.10. Transcriptome Sequencing

For transcriptome sequencing, total RNA samples were first validated for quality. Eukaryotic mRNA, with its polyA tail, was purified using Oligo(dT) magnetic beads, while prokaryotic mRNA required rRNA removal kits to obtain more informative RNA. Raw reads underwent data processing to remove low-quality sequences and adaptor contamination, resulting in clean reads. Transcriptome sequencing data analysis included three main steps: quality control of sequencing data, alignment of clean reads to the reference genome for gene classification and expression quantification, and differential expression analysis to explore gene expression changes under different conditions.

### 2.11. Subcellular Protein Fractionation

With Subcellular Protein Fractionation Kit for Cultured Cells (78840, ThermoFisher Scientific, Waltham, MA, USA), the cells were sequentially lysed to obtain functional cytoplasmic, membrane, soluble nuclear, chromatin-bound, and cytoskeletal protein fractions. Adherent cells were harvested using trypsin-EDTA and centrifuged at 500× *g* for 2–3 min. The cells were washed three times with pre-cooled PBS, and 5 × 10^6^ cells were collected in a 1.5 mL centrifuge tube, centrifuged at 500× *g* for 2–3 min, and the supernatant discarded. The cell pellet was resuspended in a specific volume of Cytoplasmic Extraction Buffer (CEB) with protease inhibitors and incubated on a 4 °C shaker for 10 min. The lysate was centrifuged at 500× *g* for 5 min at 4 °C, and the supernatant containing cytoplasmic proteins was collected. The pellet was resuspended in Membrane Extraction Buffer (MEB) with protease inhibitors, vortexed for 5 s, and incubated on a 4 °C shaker for 10 min. After centrifugation at 3000× *g* for 5 min at 4 °C, the supernatant containing membrane proteins was collected. The pellet was then resuspended in Nuclear Extraction Buffer (NEB) with protease inhibitors, vortexed for 15 s, and incubated on a 4 °C shaker for 30 min. The mixture was centrifuged at 5000× *g* for 5 min at 4 °C, and the supernatant containing soluble nuclear proteins was collected. For chromatin-bound proteins, the pellet was resuspended in chromatin-bound extraction buffer, vortexed for 15 s, and incubated at room temperature for 15 min or in a 37 °C water bath for 5 min. After vortexing for 15 s and centrifugation at 16,000× *g* for 5 min at 4 °C, the supernatant containing chromatin-bound proteins was collected. Finally, the pellet was resuspended in Cytoskeletal Protein Extraction Buffer (PEB) with protease inhibitors, vortexed for 15 s, incubated at room temperature for 10 min, and centrifuged at 16,000× *g* for 5 min. The supernatant containing cytoskeletal proteins was collected.

### 2.12. Cell Counting Kit-8 (CCK-8)

For cell proliferation and cytotoxicity detection, the CCK-8 kit (40203ES76, Yeasen Biotechnology, Shanghai, China) was used according to the manufacturer’s protocol. To create a standard curve, the number of cells in the cell suspension was first determined using a cell counting board. Cells were then seeded into a culture plate and serially diluted with culture medium to create a gradient of cell concentrations. After 2–4 h of incubation for cell attachment, CCK-8 reagent was added, and the OD value was measured after further incubation. A standard curve was plotted with cell number on the X-axis and OD value on the Y-axis to determine cell numbers in unknown samples. For cell viability detection, 100 μL of cell suspension was added to each well of a 96-well plate and pre-incubated at 37 °C with 5% CO_2_. Then, 10 μL of CCK-8 solution was added to each well and incubated for 1–4 h before measuring absorbance at 450 nm. For cell proliferation and toxicity detection, 100 μL of cell suspension was added to each well of a 96-well plate and pre-incubated for 24 h. Various concentrations of the test substance were then added, and the plate was incubated for an appropriate time. Subsequently, 10 μL of CCK-8 solution was added to each well and incubated for 1–4 h before measuring absorbance at 450 nm. The percentage of cell viability was calculated as [A(treated) − A(blank)]/[A(control) − A(blank)] × 100.

### 2.13. Imaging

Immunolabeled slides, including paraffin sections, and cell coverslips, were imaged by confocal microscopy (Dragonfly Spinning Disc confocal microscope driven by Fusion Software (version 2.4), Andor Technology, Oxford Instruments, Oxford, UK). Projection images were then processed and analyzed using Bitplane Imaris (version 9.7) software.

### 2.14. Statistical Analysis

Data were analyzed using SPSS 21.0 and GraphPad Prism 8.0. Data are presented as the mean ± standard deviation (SD) or mean ± standard error (SEM). The numerical results between 2 groups were compared by two-tailed Student’s *t* test, while one-way ANOVA followed by Dunnett’s test was used for comparisons among three or more groups. A *p* value of less than 0.05 was considered significant.

## 3. Results

### 3.1. Expression of ROCK in Leydig Cells of Mammalian Testes

**Figure 1:**
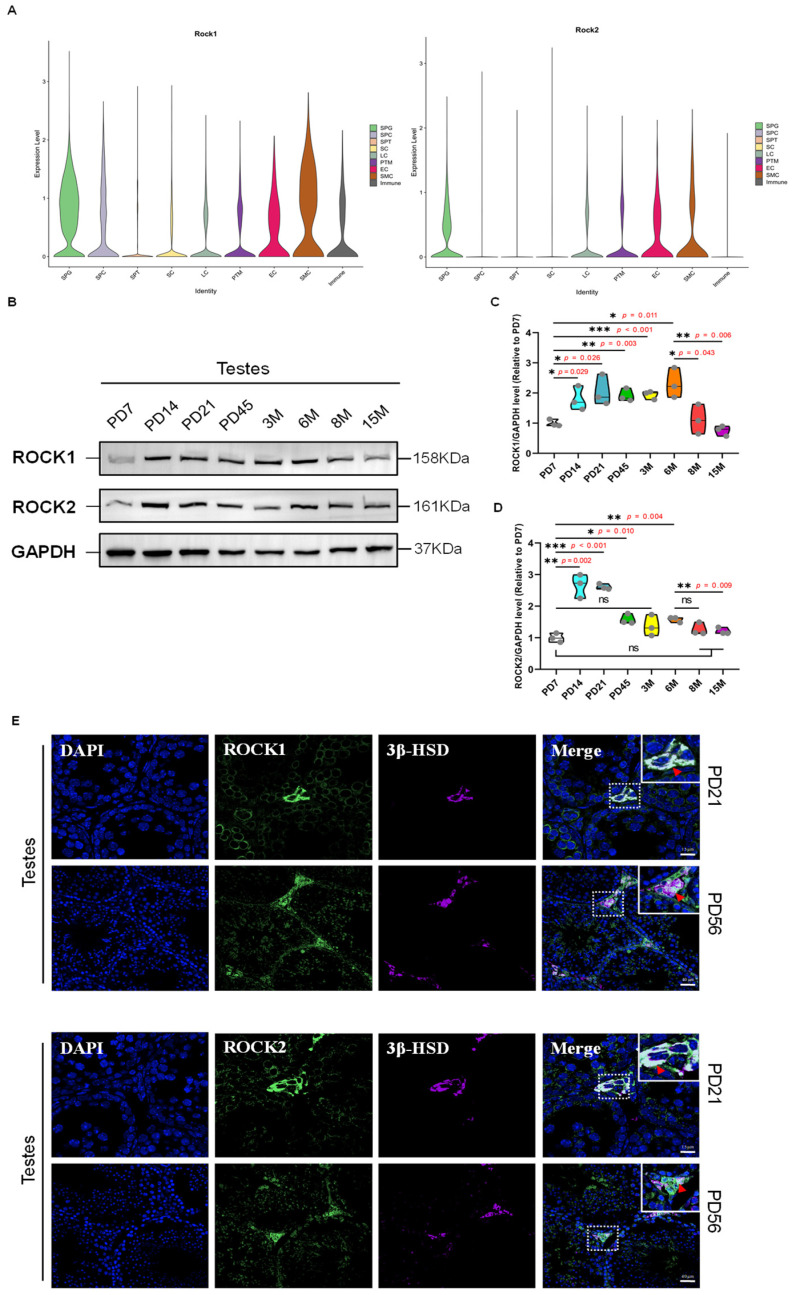
ROCK Expression and Localization in Mouse Testes. (**A**) Violin plots indicate the expression levels of ROCK1 and ROCK2 in testicular Leydig cells. Transcriptomic data were obtained from the Male Health Atlas (http://malehealthatlas.cn/, accessed on 6 March 2024.). SPG: spermatogonia; SPC: spermatocytes; SPT: spermatids/sperms; SC: Sertoli cells; LC: Leydig cells; PTM: peritubular myoid cells; EC: Endothelial cells; SMC: vascular smooth muscle cells; Immune: Immune cells. (**B**) Western Blot analysis shows the expression of ROCK1 and ROCK2 in the testes from different developmental stages, with a noted decrease in ROCK1 expression in the testes of aged mice, using GAPDH as a loading control. (**C**) Quantification of results of Western Blot analysis of ROCK1 from (**B**), normalized to PD7 (*n* = 3). Statistical significance was determined by unpaired two-tailed *t*-test. * *p* < 0.05; ** *p* < 0.01; *** *p* < 0.001. (**D**) Quantification of results of Western Blot analysis of ROCK2 from (**B**), normalized to PD7 (*n* = 3). Statistical significance was determined by unpaired two-tailed *t*-test. ns: not significant; * *p* < 0.05; ** *p* < 0.01; *** *p* < 0.001. (**E**) Immunofluorescence results on paraffin sections demonstrate that ROCK1 and ROCK2 (green) are primarily localized in 3β-HSD-positive (purple) Leydig cells (indicated by red triangles) in the testes of PD21 (top row) and PD56 (bottom row) mice, with nuclei stained by DAPI (blue). The dashed boxed areas are magnified. Scale bars: PD21 (top row): 15 µm; PD56 (bottom row): 40 µm.

To investigate the roles of ROCK in Leydig cell functions, we analyzed the expression of ROCK1 and ROCK2 in various testicular cell types using single-cell transcriptomic data from the Male Health Atlas database [20]. Analysis of single-cell transcriptomic data from adult human and mouse testes (Male Health Atlas) showed that ROCK1 and ROCK2 are highly expressed in vascular smooth muscle cells and endothelial cells. Among testicular somatic cells, both isoforms exhibited moderately high expression in Leydig cells, clearly higher than in Sertoli cells. Within the germ cell lineage, ROCKs showed relatively high expression in spermatogonia, with lower levels in spermatocytes and the lowest in spermatids (Figure 1A and Figure S1). These patterns suggest distinct roles for ROCK isoforms in testicular vascular structure, steroidogenic regulation, and early stages of spermatogenesis. This indicates a potential involvement of ROCK proteins in the biological processes of Leydig cells, possibly including maintenance of the spermatogenic microenvironment and regulation of reproductive endocrinology. Furthermore, we analyzed the expression of ROCK1 and ROCK2 in testes from mice at various developmental stages. Western Blot results demonstrated a significant upregulation of ROCK1 and ROCK2 expression from early postnatal days (PD14 to PD21), persisting through multiple stages of development. ROCK1 expression was notably higher in the testes of mice during puberty (PD45) and adulthood (3M, 6M), but decreased in aged mice (15M) (Figure 1B–D). Additionally, we used the specific antibody targeting 3β-HSD, which is a key enzyme involved in testosterone synthesis, to label Leydig cells in the interstitial region of testes. Immunofluorescence analysis showed that ROCK1 and ROCK2 proteins are obviously expressed in interstitial cells of the testis, and colocalization with 3β-HSD indicated their presence in Leydig cells (Figure 1E). These findings support the notion that ROCK1 and ROCK2 are involved in Leydig cell function, and the observed downregulation of ROCK1 with aging may correlate with reduced testicular function and reproductive senescence.

### 3.2. Effects of ROCK Inhibitor Y-27632 on Cytoskeletal Organization in TM3 Cells

**Figure 2:**
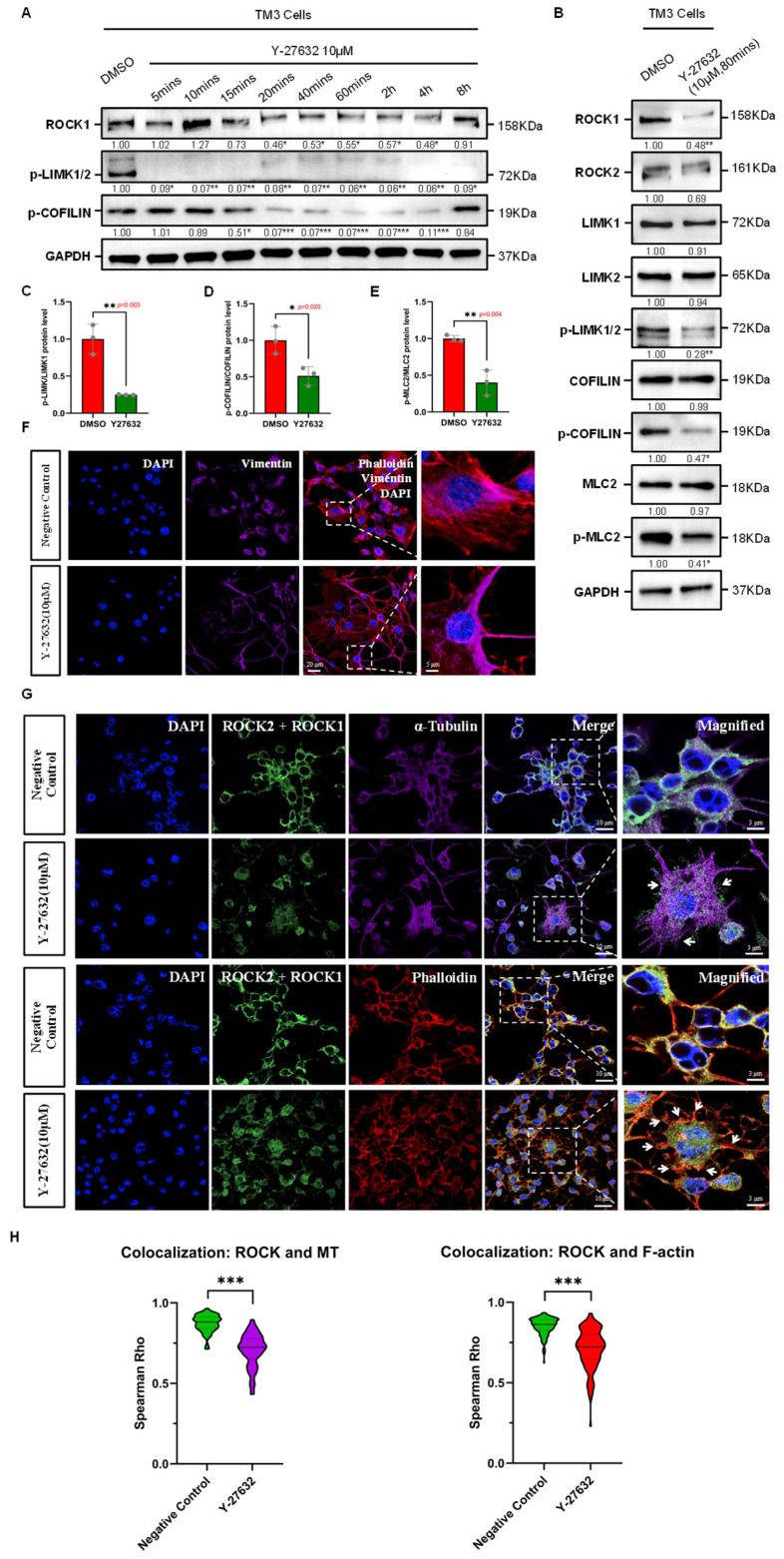
Effects of ROCK-i on Cytoskeletal Remodeling in Leydig Cells. (**A**) Effective duration of Y-27632 treatment. Western Blot results show that Y-27632 significantly inhibits ROCK activity in TM3 cells within the effective duration, with a notable decrease in phosphorylation levels of downstream substrates LIMK and COFILIN. The effective duration ranges from 15 min to 4 h, with GAPDH used as a loading control. Data are mean of *n* = 3. Values were normalized to the DMSO group. Statistical significance was determined by unpaired two-tailed *t*-test. * *p* < 0.05; ** *p* < 0.01; *** *p* < 0.001. (**B**) The phosphorylation levels of cytoskeletal remodeling regulators LIMK, COFILIN, and MLC2 in TM3 cells are significantly reduced by ROCK-i treatment, with GAPDH used as a loading control. Data are mean of *n* = 3. Values were normalized to the DMSO group. Statistical significance was determined by unpaired two-tailed *t*-test. * *p* < 0.05; ** *p* < 0.01. (**C**) Quantification of phospho-LIMK/LIMK1 protein ratio from (**B**), normalized to DMSO group (*n* = 3). The ratio was significantly reduced in Y-27632 group (*p* = 0.003). Statistical significance was determined by unpaired two-tailed *t*-test. ** *p* < 0.01. (**D**) Quantification of phospho-COFILIN/COFILIN protein ratio from (**B**), normalized to DMSO group (*n* = 3). The ratio was significantly reduced in Y-27632 group (*p* = 0.020). Statistical significance was determined by unpaired two-tailed *t*-test. * *p* < 0.05. (**E**) Quantification of phospho-MLC2/MLC2 protein ratio from (**B**), normalized to DMSO group (*n* = 3). The ratio was significantly reduced in Y-27632 group (*p* = 0.004). Statistical significance was determined by unpaired two-tailed *t*-test. ** *p* < 0.01. (**F**) Effects of ROCK-i on the intermediate filament cytoskeletal structure in TM3 cells. Red fluorescence indicates Phalloidin-labeled microfilaments; purple fluorescence indicates Vimentin-labeled intermediate filaments; nuclei are stained with DAPI (blue). The dashed box areas are magnified. Scale bars: original areas: 20 µm; magnified areas: 5 µm. (**G**) Effects of ROCK-i on the microfilament and microtubule cytoskeletal structures in TM3 cells. Green fluorescence indicates ROCK1/2 localization; red fluorescence indicates Phalloidin-labeled microfilaments; purple fluorescence indicates α-Tubulin-labeled microtubules; nuclei are stained with DAPI (blue). The dashed box areas are magnified. Arrows in the top row indicate abnormally aggregated microtubules, while arrows in the bottom row indicate abnormally aggregated microfilaments at cell junctions. Scale bars: original areas: 10 µm; magnified areas: 3 µm. (**H**) Violin plots showing Spearman correlation coefficients (Spearman Rho) quantifying the colocalization of ROCK1/2 with microtubules and F-actin in TM3 cells. Each group included 50 cells per replicate (*n* = 3). Y-27632 treatment significantly reduced the colocalization of ROCK1/2 with both microtubules (Negative Control: 0.8724 ± 0.0521; Y-27632: 0.7055 ± 0.1029; *p* < 0.001, Mann–Whitney U test) and F-actin (Negative Control: 0.8509 ± 0.0536; Y-27632: 0.7110 ± 0.1226; *p* < 0.001, Mann–Whitney U test). Data are presented as the mean ± SD. *** *p* < 0.001.

The discovery and application of specific ROCK inhibitors are crucial for exploring the biological implications of the Rho/ROCK pathway. ROCK inhibitors are known to suppress ROCK activity by competitively inhibiting the binding of Rho GTPases to ROCK and the ATP-dependent phosphorylation of substrates [21,22]. Currently, more than 170 small molecular compounds have been identified as ROCK inhibitors. Among them, Y-27632, a potent and specific inhibitor of ROCK, has been widely used in both in vivo and in vitro studies targeting ROCK [8]. Y-27632 has inhibition constants (Ki) of 0.22 µM for ROCK1 and 0.30 µM for ROCK2. In vitro studies indicate that treatment with Y-27632 can alter the phosphorylation state of cytoskeletal-related proteins such as Myosin Phosphatase Subunit 1 (MYPT1), affecting various cytoskeletal-related biological events, including cell migration [6]. The duration of protein kinase inhibitor effects may vary among different types of tissues and cells. We first tested the effective duration of Y-27632 in TM3 cells by assessing the phosphorylation levels of ROCK substrates at various time points during drug treatment via Western blot, observing changes in ROCK activity. The results show that from 5 min post-treatment with Y-27632, the expression level of p-LIMK significantly decreased, and as a downstream substrate of LIMK, the phosphorylation level of COFILIN began to decrease after 15 min of treatment, continuing up to 4 h and recovering after 8 h (Figure 2A). Given the immediate function of COFILIN in severing F-actin during actin filament remodeling, our observations suggest that Y-27632 induces temporal suppression of ROCK in TM3 cells from 15 min to 4 h. The diminishing effect of Y-27632 post-treatment might be attributed to the drug’s metabolic or dissociation rates.

To explore the impact of ROCK-i on cytoskeletal remodeling in TM3 cells, we first examined the changes in phosphorylation levels of cytoskeletal remodeling factors LIMK, COFILIN, and MLC2 under the influence of Y-27632. Western Blot results indicated that during the effective duration of Y-27632, the phosphorylation levels of LIMK, COFILIN, and MLC2 in TM3 cells were significantly reduced, while their total protein expression levels did not show significant changes (Figure 2B–E and (Figure S2), and F-ACTIN expression levels decreased (Figure S2). Beyond the effective duration of Y-27632, the phosphorylation levels of LIMK, COFILIN, and MLC2 recovered, and F-ACTIN expression levels increased (Figure S2). These findings indicate that ROCK-i modulates the phosphorylation states of cytoskeletal remodeling factors and is associated with changes in F-ACTIN expression over time. Additionally, through immunofluorescence experiments in cells, we observed changes in the cytoskeletal structure of TM3 cells before and after treatment with Y-27632. In TM3 cells, we also observed cytoskeletal remodeling of intermediate filaments (marked by Vimentin) around the cell nucleus, which showed a distinct polarity in distribution, mainly concentrated on specific sides of the cell and at cell junction areas (Figure 2F). As shown in Figure 2G, ROCK1/2 was mainly localized in the cytoskeletal regions of the cytoplasm of TM3 cells and showed colocalization with α-Tubulin (marker of microtubule) and Phalloidin (marker of actin filament). After Y-27632 treatment, the localization of ROCK1/2 in regions of the actin and microtubule cytoskeleton decreased, with visibly abnormal aggregation of thick coalesced microtubule structures and fewer stress fiber-like actin structures, along with abnormal aggregations at the cell junctions (Figure 2G,H). These observations collectively suggest that ROCK inhibition, within its effective window, correlates with broad alterations in cytoskeletal structures, including microtubules, actin filaments, and intermediate filaments in TM3 cells, and disrupts the function and remodeling process of the actin cytoskeleton by inhibiting the phosphorylation of cytoskeletal regulatory factors such as LIMK, COFILIN, and MLC2.

### 3.3. Evaluation of Y-27632 on Proliferation and Cytotoxicity in TM3 Cells

To assess the bioactivity and safety of Y-27632 in TM3 cells, we first conducted Cell Counting Kit-8 (CCK-8) assays to evaluate its effects on cell proliferation and cytotoxicity, setting up multiple replicates related to time and drug concentration gradients. The CCK-8 assay results indicated that during the effective duration of Y-27632 (15 min to 4 h), there was no significant change in TM3 cell viability; within 24 h of Y-27632 treatment, there was no apparent proliferation toxicity, and it was not significantly correlated with the concentration of the drug (Figure S3A). These findings suggest that, under our experimental conditions, the influence of Y-27632 on TM3 cell viability may be more sensitive to exposure duration than to concentration. Additionally, results from immunofluorescence staining of TM3 cells demonstrated that 12 h post Y-27632 treatment, there was a significant increase in the number of Ki-67 positive cells, a marker of cell proliferation, and a decrease in the number of cells positive for cleaved Caspase-3, an apoptosis-related protein (Figure S3B). These changes suggest that Y-27632 treatment may be linked to enhanced proliferation and reduced apoptosis in TM3 cells. The discrepancy between the results of CCK-8 assay and the immunostaining could be due to the different sensitivities of these assays in detecting changes in cell proliferation. In summary, these data indicate that Y-27632 does not exert overt cytotoxicity in TM3 cells within 24 h of exposure and may modulate cell proliferation and survival pathways.

### 3.4. hCG Stimulation Is Associated with ROCK1 Activation in TM3 Cells

**Figure 3:**
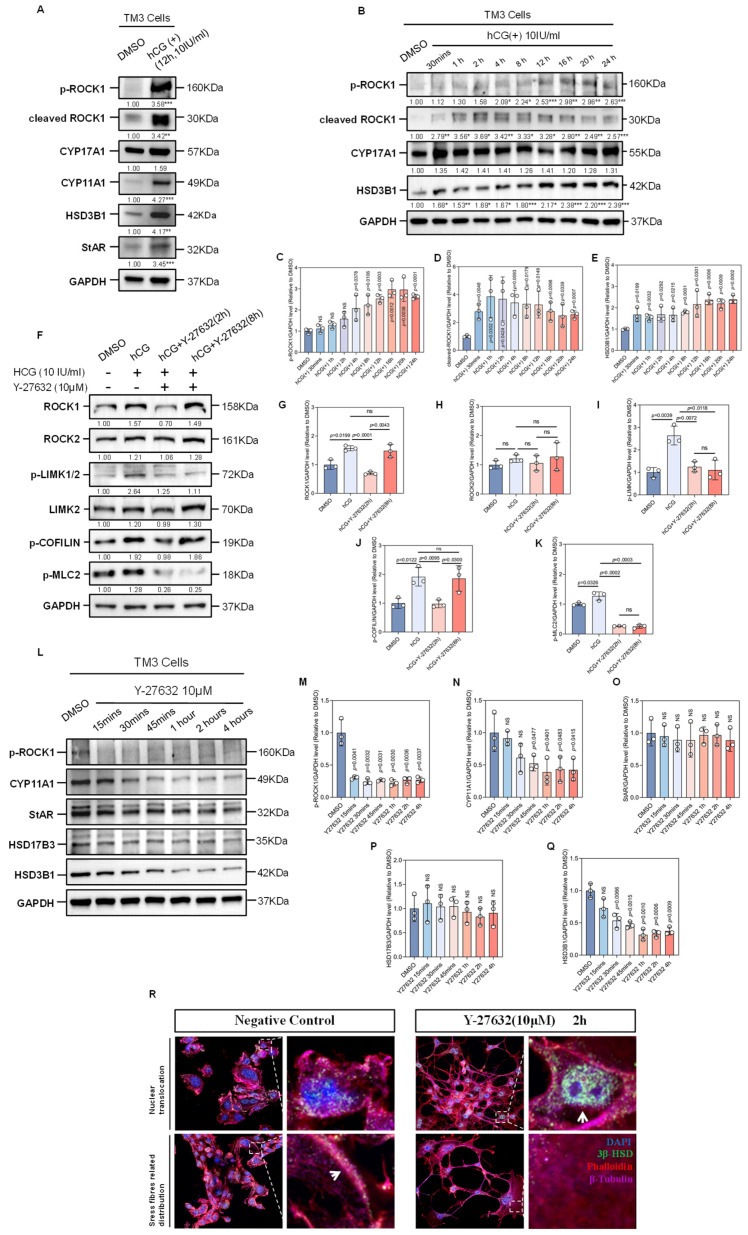
Effects of hCG and ROCK-i on the Expression of Factors of Microfilament Remodeling and Testosterone Synthesis-Related Enzymes. (**A**) Western Blot analysis shows the expression of p-ROCK1, cleaved ROCK1, CYP11A1, CYP17A1, HSD3B1, and StAR in TM3 cells before and after hCG treatment, with GAPDH as a loading control. Data are mean of *n* = 3. Values were normalized to the DMSO group. Statistical significance was determined by unpaired two-tailed *t*-test. ** *p* < 0.01; *** *p* < 0.001. (**B**) Western Blot analysis shows the changes in the expression of p-ROCK1, cleaved ROCK1, CYP17A1, and HSD3B1 over different durations of hCG treatment, with GAPDH as a loading control. Data are mean of *n* = 3. Values were normalized to the DMSO group. Statistical significance was determined by unpaired two-tailed *t*-test. * *p* < 0.05; ** *p* < 0.01; *** *p* < 0.001. (**C**) Quantification of results of Western Blot analysis of p-ROCK1 from (**B**), normalized to DMSO (*n* = 3). Statistical significance was determined by unpaired two-tailed *t*-test between hCG group with certain duration and DMSO group. (**D**) Quantification of results of Western Blot analysis of cleaved ROCK1 from (**B**), normalized to DMSO (*n* = 3). Statistical significance was determined by unpaired two-tailed *t*-test between hCG group with certain duration and DMSO group. (**E**) Quantification of results of Western Blot analysis of HSD3B1 from (**B**), normalized to DMSO (*n* = 3). Statistical significance was determined by unpaired two-tailed *t*-test between hCG group with certain duration and DMSO group. (**F**) Western Blot analysis shows the changes in the expression of ROCK1, ROCK2, p-LIMK1/2, LIMK2, p-COFILIN, and p-MLC2 in TM3 cells following hCG stimulation and Y-27632 treatment over time, with Y-27632 treatment applied after 12 h of hCG stimulation, using GAPDH as a loading control. Data are mean of *n* = 3. Values were normalized to the DMSO group. (**G**) Quantification of results of Western Blot analysis of ROCK1 from (**F**), normalized to DMSO (*n* = 3). ns: not significant. (**H**) Quantification of results of Western Blot analysis of ROCK2 from (**F**), normalized to DMSO (*n* = 3). ns: not significant. (**I**) Quantification of results of Western Blot analysis of p-LIMK from (**F**), normalized to DMSO (*n* = 3). ns: not significant. (**J**) Quantification of results of Western Blot analysis of p-COFILIN from (**F**), normalized to DMSO (*n* = 3). ns: not significant. (**K**) Quantification of results of Western Blot analysis of p-MLC2 from (**F**), normalized to DMSO (*n* = 3). ns: not significant. (**L**) Western Blot analysis shows the changes in the expression of p-ROCK1, CYP11A1, StAR, HSD17B3, and HSD3B1 in TM3 cells over different durations of Y-27632 treatment, with GAPDH as a loading control. (**M**) Quantification of results of Western Blot analysis of p-ROCK1 from (**L**), normalized to DMSO (*n* = 3). (**N**) Quantification of results of Western Blot analysis of CYP11A1 from (**L**), normalized to DMSO (*n* = 3). ns: not significant. (**O**) Quantification of results of Western Blot analysis of StAR from (**L**), normalized to DMSO (*n* = 3). ns: not significant. (**P**) Quantification of results of Western Blot analysis of HSD17B3 from (**L**), normalized to DMSO (*n* = 3). ns: not significant. (**Q**) Quantification of results of Western Blot analysis of HSD3B1 from (**L**), normalized to DMSO (*n* = 3). ns: not significant. (**R**) Immunofluorescence of cell coverslips shows the changes in the localization pattern of 3β-HSD in TM3 cells. Red fluorescence indicates Phalloidin-labeled microfilaments; purple fluorescence indicates β-Tubulin-labeled microtubules; green fluorescence indicates 3β-HSD; nuclei are stained with DAPI (blue). The dashed box areas are magnified. Arrows in the left panel indicate 3β-HSD distributed along stress fibers, while arrows in the right panel indicate nuclear translocation of 3β-HSD. Scale bars: original areas: 15 µm; magnified areas: 2 µm.

In mammals, testosterone synthesis is initiated in Leydig cells via the LHCGR membrane receptor, which responds to LH or hCG hormone signals, activating and transmitting the cAMP-PKA dependent signaling pathway. The LH (hCG)-LHCGR signaling axis coordinates multiple biological events, such as the transport of testosterone synthesis precursors and steroid biosynthesis, thereby promoting testosterone synthesis [23]. Based on previous results showing ROCK’s involvement in actin cytoskeleton remodeling, we hypothesized that ROCK is activated by the LH (hCG)-LHCGR signaling pathway to mediate its regulatory role in the intracellular traffic events during testosterone synthesis. To validate this hypothesis, we measured the levels of phosphorylated ROCK1 and the expression of cleaved ROCK1 in TM3 cells, both markers of ROCK1 activation [6,24], before and after induction with exogenous hCG. Western Blot results demonstrated a significant increase in the expression levels of p-ROCK1 and cleaved ROCK1 proteins under hCG stimulation, along with a concurrent increase in the expression of key enzymes involved in the process of testosterone synthesis, CYP11A1 and HSD3B1 (Figure 3A). Additionally, we observed a time-dependent increase in the expression levels of p-ROCK1, cleaved ROCK1, and HSD3B1 in TM3 cells induced with hCG, particularly after 12–16 h of treatment, when the expression levels were relatively high (Figure 3B–E). These findings suggest that hCG stimulation correlates with ROCK1 activation and upregulation of steroidogenic enzymes in TM3 cells.

### 3.5. ROCK-i Reverses hCG-Induced F-Actin Remodeling

We next explored the impact of the hCG-LHCGR signaling pathway on the cytoskeletal remodeling of Leydig cells. Results from protein immunoblotting experiments demostrated that post-hCG induction, the phosphorylation levels of LIMK, COFILIN, and MLC2 increased within the cells. These changes were temporally associated with hCG stimulation, suggesting activation of signaling components linked to actin cytoskeleton dynamics. Treatment with Y-27632 for two hours following the hCG induction in Leydig cells returned the phosphorylation levels of these regulators of F-actin remodeling to baseline (Figure 3F–K), indicating that ROCK activity is required to sustain hCG-associated phosphorylation events, although the precise signaling hierarchy remains to be clarified. These data suggest that hCG-LHCGR signaling may influence the remodeling of the actin cytoskeleton through pathways involving LIMK, COFILIN, and MLC2 phosphorylation, potentially mediated in part by ROCKs. Conversely, ROCK-i reversed the effects of hCG signaling on these actin cytoskeleton regulatory factors. Additionally, during its effective duration, Y-27632 significantly reduced the expression levels of p-ROCK1, CYP11A1, and HSD3B1 in the TM3 cells. The observed suppression of p-ROCK1 and key steroidogenic enzymes by Y-27632 contrasts with the upregulation induced by hCG, supporting a functional antagonism between ROCK inhibition and hCG signaling in these pathways (Figure 3L–Q).

HSD3B1 is the primary isoform of the steroid reductase 3β-HSD in mice, predominantly localized in the smooth endoplasmic reticulum of Leydig cells, where it is responsible for converting pregnenolone to progesterone, playing a crucial role in the testosterone synthesis pathway [25]. Immunofluorescence experiments revealed that ROCK inhibition via Y-27632 was accompanied by a redistribution of 3β-HSD, with an increased nuclear-like localization and reduced colocalization with F-actin structures in the cytoplasm (Figure 3R). While the mechanism underlying this shift remains to be clarified, these observations raise the possibility that cytoskeletal architecture may influence the subcellular trafficking or anchoring of steroidogenic enzymes.

**Figure 4:**
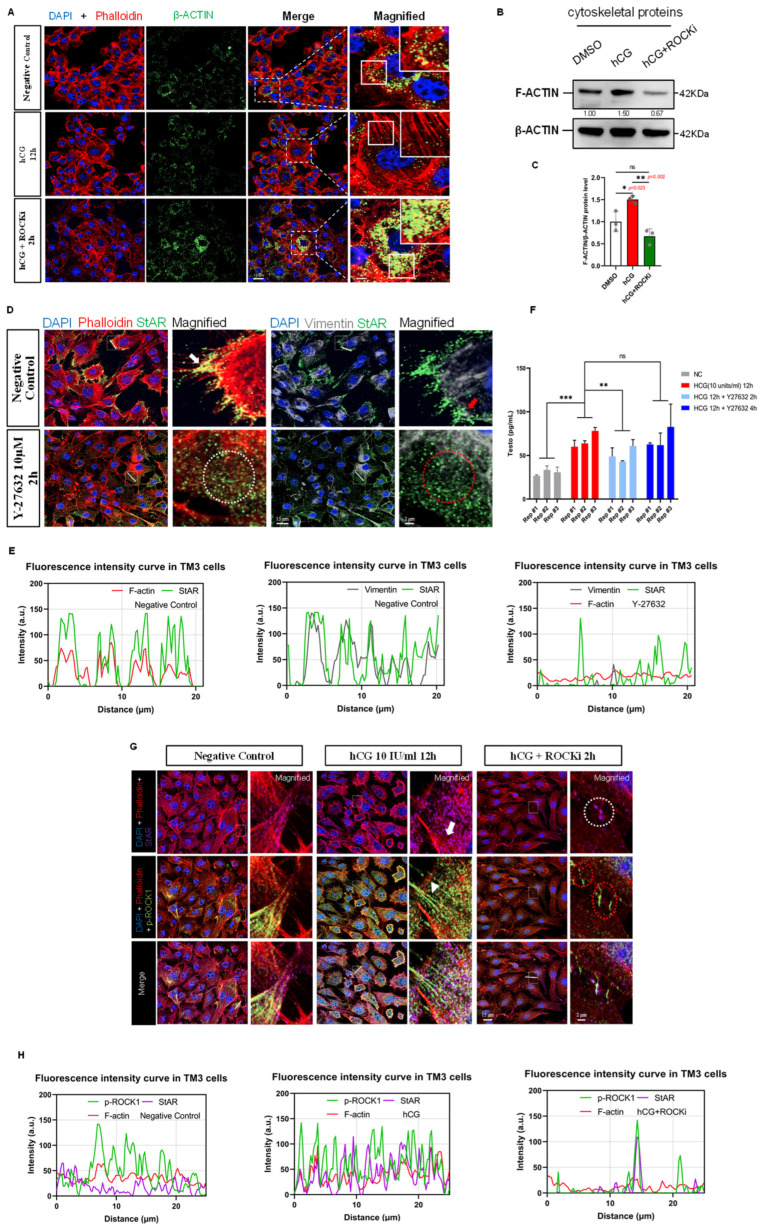
ROCK-i Reverses hCG-Induced F-actin Remodeling and Testosterone Synthesis in Leydig Cells. (**A**) Immunofluorescence of cell coverslips shows changes in F-actin stress fiber structures in TM3 cells. Y-27632 treatment was applied following 12 h of hCG stimulation. Red fluorescence indicates Phalloidin-labeled microfilaments; green fluorescence indicates β-ACTIN monomeric actin; nuclei are stained with DAPI (blue). The dashed box areas are magnified. Scale bars: original areas: 15 µm; magnified areas: 5 µm. (**B**) Western Blot analysis shows changes in F-ACTIN expression in cytoskeletal protein fractions in response to hCG stimulation and Y-27632 treatment over time. Y-27632 treatment was applied following 12 h of hCG stimulation, with β-ACTIN used as a loading control. Data are mean of *n* = 3. Values were normalized to the DMSO group. (**C**) Quantification of F-ACTIN/β-ACTIN protein ratio from (**B**), normalized to DMSO (*n* = 3). ns: not significant. * *p* < 0.05; ** *p* < 0.01. (**D**) Immunofluorescence of cell coverslips shows the distribution changes in StAR (green), Phalloidin (red), and Vimentin (gray) in TM3 cells. Nuclei are stained with DAPI (blue). The dashed box areas are magnified. White arrows indicate StAR localization in microfilament stress fiber regions; red arrows indicate StAR localization with Vimentin-labeled intermediate filaments; white dashed circles indicate StAR dispersed distribution influenced by ROCK-i, unrelated to F-actin; red dashed circles indicate StAR distribution unrelated to Vimentin; white lines indicate the regions selected for colocalization analysis. Scale bars: original areas: 15 µm; magnified areas: 2 µm. (**E**) Fluorescence intensity profiles of StAR (green), F-actin (red), and Vimentin (gray) in TM3 cells. In the Negative Control group, StAR showed colocalization with F-actin (Rho = 0.686) and Vimentin (Rho = 0.488). After Y-27632 treatment, this colocalization was significantly reduced (Rho = 0.140 and 0.289, respectively), indicating cytoskeletal disruption. (**F**) ROCK-i inhibits hCG-induced testosterone synthesis and secretion. Testosterone levels were 30.30 ± 1.950 pg/mL in the negative control group, 67.37 ± 5.521 pg/mL in the hCG 12 h group, 48.60 ± 3.820 pg/mL in the hCG 12 h + Y27632 2 h group, and 69.07 ± 6.871 pg/mL in the hCG 12 h + Y27632 4 h group (*n* = 3, data presented as mean ± SEM). ns: not significant; ** *p* < 0.01; *** *p* < 0.001. (**G**) Distribution of p-ROCK1 (green), StAR (purple), and Phalloidin (red) in TM3 cells. Nuclei are stained with DAPI (blue). Y-27632 treatment was applied following 12 h of hCG stimulation. The dashed box areas are magnified. White arrows indicate StAR localization in microfilament stress fiber regions; white triangles indicate p-ROCK1 localization in microfilament stress fiber regions; white dashed circles indicate abnormal StAR distribution at cell junctions; red dashed circles indicate abnormal p-ROCK1 distribution at cell junctions; white lines indicate the regions selected for colocalization analysis. Scale bars: original areas: 15 µm; magnified areas: 2 µm. (**H**) Fluorescence intensity profiles of StAR (purple), F-actin (red), and p-ROCK1 (green) in TM3 cells. In the hCG group, F-actin showed colocalization with StAR (Rho = 0.483) and p-ROCK1 (Rho = 0.430). In contrast, colocalization was reduced in the hCG + ROCKi group (Rho = 0.126 and 0.196, respectively), indicating that ROCK inhibition disrupts their spatial association.

Another set of results from immunofluorescence experiments showed that treatment with exogenous hCG and a ROCK inhibitor led to changes in the organization of the F-actin structure in Leydig cells: after hCG treatment, robust filamentous Phalloidin fluorescence signals were observed in the cytoplasm, indicating the assembly of F-actin stress fibers in Leydig cells; In contrast, subsequent treatment with ROCK inhibitor led to a loss of filamentous signal and a relative increase in diffuse β-actin staining, consistent with F-actin disassembly (Figure 4A).

With the utilization of subcellular protein fractionation techniques, we enriched the cytoskeletal protein fractions of TM3 cells. Using β-ACTIN as a loading control, we observed elevated F-ACTIN levels in hCG-treated cells and a reduction in Y-27632-treated cells (Figure 4B,C), aligning with the immunofluorescence data. Together, these findings suggest that hCG promotes F-actin polymerization in Leydig cells, potentially via activation of ROCK signaling, whereas ROCK inhibition reverses this remodeling. Actin cytoskeletal dynamics, modulated by ROCK signaling, are closely associated with changes in the localization of key steroidogenic enzymes and possibly contribute to the cellular machinery governing testosterone biosynthesis.

### 3.6. ROCK-i Reduces Testosterone Synthesis in Leydig Cells

To evaluate the impact of ROCK inhibition on testosterone biosynthesis in TM3 cells, we employed ELISA techniques to measure the testosterone levels in the cell culture medium. After obtaining a suitable standard curve (Figure S4A), we observed that hCG signaling significantly enhanced the synthesis and secretion of testosterone in Leydig cells. At the 6 h and 12 h time points post-hCG treatment, the testosterone content in the culture medium was notably higher than that in the negative control group, showing a positive correlation with the duration of hCG stimulation within the 0–12 h window (Figure S4B).

Furthermore, we examined the effect of ROCK inhibition on hCG-induced testosterone production. At the 2 h time point post-Y-27632 treatment—corresponding to the effective inhibition window—testosterone levels in the medium were significantly reduced compared to the hCG group. However, at 4 h post-treatment, testosterone secretion increased and showed no significant difference from the hCG-treated group, suggesting a possible compensatory effect or metabolic inactivation of the inhibitor (Figure 4F). It is plausible that ROCK inhibition perturbs cytoskeletal organization or interferes with signaling intermediates required for steroidogenic enzyme function or cholesterol transport. Taken together, the data support the interpretation that Y-27632 interferes with hCG-induced testosterone production in a time-sensitive manner, with the most pronounced effect observed during its pharmacologically active window.

### 3.7. ROCK-i Alters the Localization of Cholesterol Transport-Related Proteins in the Cytoskeleton Region

StAR mediates cholesterol transport to the inner mitochondrial membrane, a rate-limiting step in testosterone biosynthesis. Evidence suggests that this process is closely associated with cytoskeletal-mediated organelle transport, including interactions between lipid droplets and mitochondria, which may be regulated by the cytoskeleton [5]. To explore the mechanism by which ROCK inhibition interferes with testosterone biosynthesis, we first examined the effect of Y-27632 treatment on the subcellular localization of StAR. Immunofluorescence results showed that in Leydig cells, StAR is primarily located in the peripheral regions of the cytoplasm and co-localizes with Phalloidin-labeled F-actin signals, with clusters of StAR observed in the actin stress fiber regions (Figure 4D,E). Additionally, the distribution of StAR was adjacent to regions marked by Vimentin-labeled intermediate filaments (Figure 4D,E), indicating that intermediate filament structures might be related to the biological functions of StAR.

Following treatment with ROCK-i, we observed a loss of specific distribution of StAR in Leydig cells. Immunofluorescence observations indicated that StAR signals were diffusely distributed throughout the cytoplasm and their localization was not associated with the distribution of disorganized actin and intermediate filaments (Figure 4D,E), suggesting that ROCK-i disrupts the cytoskeletal framework supporting StAR localization, potentially impairing its functional positioning. Based on these observations, we further co-stained p-ROCK1, StAR, and Phalloidin, and found that hCG signaling induced the formation of ring-like specialized F-actin structures around Leydig cells, with a substantial co-localization of p-ROCK1 and StAR at actin stress fibers (Figure 4G,H). After 2 h of ROCK-i treatment in hCG-stimulated TM3 cells, the p-ROCK1 fluorescence signals significantly decreased, accompanying the disassembly of the actin structure, with StAR and p-ROCK1 observed only at cell junctions in stacked distributions (Figure 4G,H). Taken together, these results reveal a strong spatial association between ROCK1 activity, F-actin architecture, and the positioning of StAR in TM3 cells. Our data support a model in which the cytoskeleton—modulated by ROCK signaling—acts as a structural determinant of StAR localization. Therefore, the redistribution of StAR upon ROCK inhibition may contribute to, rather than directly cause, the reduction in testosterone biosynthesis.

### 3.8. ROCK-i Disrupts Cholesterol Transport to Mitochondria

**Figure 5:**
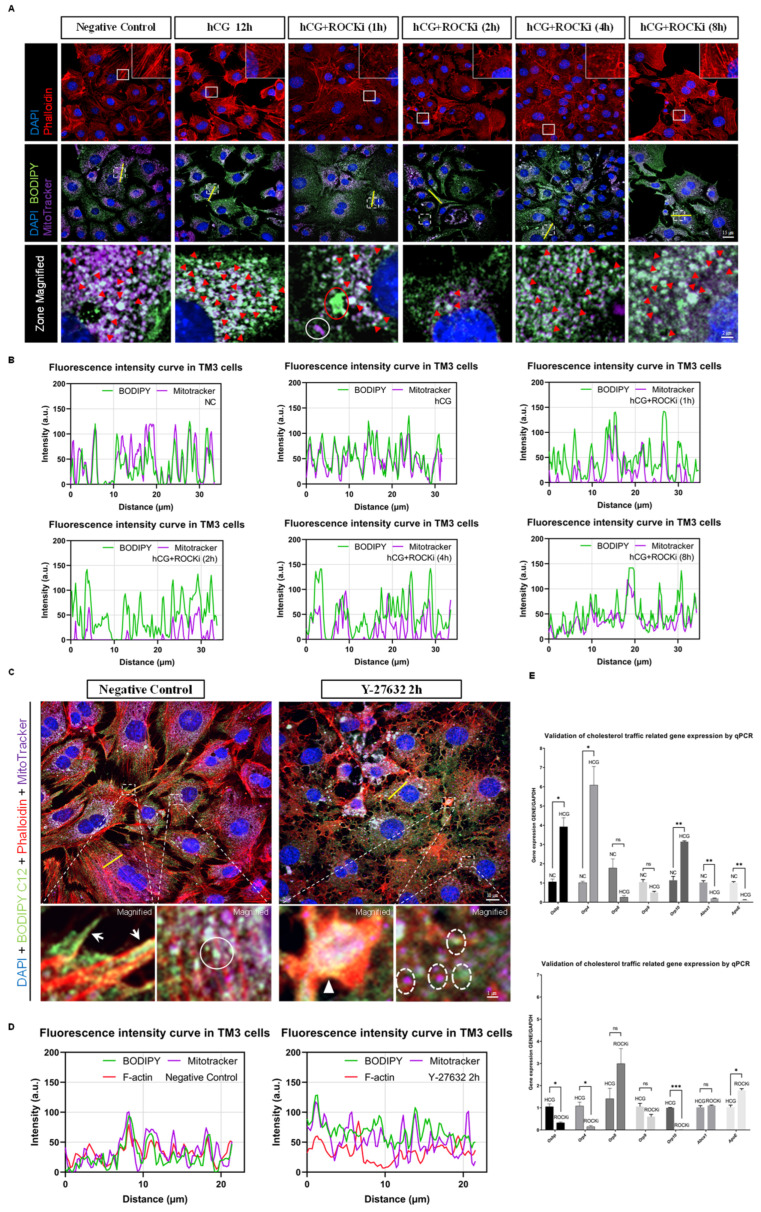
ROCK-i Affects Cholesterol Transport to Mitochondria in Leydig Cells. (**A**) Changes in the colocalization of cholesterol and mitochondria in TM3 cells. Free cholesterol is labeled with the CholEsteryl BODIPY fluorescent probe (green), mitochondria are labeled with the MitoTracker fluorescent probe (purple), and microfilaments are labeled with Phalloidin (red). Nuclei are stained with DAPI (blue). The dashed box areas are magnified. Red arrows indicate colocalized fluorescent signal points; white circles indicate mitochondrial clustering; red circles indicate cholesterol accumulation; yellow lines indicate the regions selected for colocalization analysis. Scale bars: original areas: 15 µm; magnified areas: 2 µm. (**B**) Quantification of cholesterol–mitochondria colocalization in TM3 cells. Colocalization between BODIPY-labeled cholesterol and MitoTracker-labeled mitochondria was quantified across six groups: Negative Control, HCG 12 h, HCG + ROCKi (1 h, 2 h, 4 h, 8 h). (**C**) Microfilament-mediated transport of cholesterol to mitochondria. Free cholesterol is labeled with the CholEsteryl BODIPY fluorescent probe (green), mitochondria are labeled with the MitoTracker fluorescent probe (purple), and microfilaments are labeled with Phalloidin (red). Nuclei are stained with DAPI (blue). The dashed box areas are magnified. White arrows indicate cholesterol localization in the microfilament regions; white circles indicate colocalization of cholesterol and mitochondria; white triangles indicate abnormally aggregated fluorescent signals at cell junctions; white dashed circles indicate the free state of cholesterol and mitochondria; yellow lines indicate the regions selected for colocalization analysis. Scale bars: original areas: 10 µm; magnified areas: 1 µm. (**D**) Fluorescence intensity profiles of BODIPY (green), F-actin (red), and MitoTracker (purple) in TM3 cells across Negative Control and Y-27632 2h groups. (**E**) qPCR results showing the mRNA expression levels of cholesterol transport-related genes, including *Osbp*, *Orp4*, *Orp8*, *Orp9*, *Orp10*, *Abca1*, and *ApoE*, in TM3 cells under three experimental conditions: NC (Negative Control), HCG (hCG-treated 12 h), and ROCKi (Y-27632-treated 2 h following hCG treatment). Gene expression was normalized to Gapdh and presented as mean ± SEM (*n* = 3), with expression in the NC group set to 1. For *Orp8* and *Orp9*, no significant differences were observed between NC, HCG and ROCKi groups. *Osbp*, *Orp4* and *Orp10* were significantly upregulated in HCG group compared to NC group, which expression were downregulated in ROCKi group compared to HCG group. In the HCG group, expression of *Abca1* and *ApoE* was significantly decreased compared to NC, whereas these genes were either unchanged or significantly upregulated in the ROCKi group. Statistical significance was determined by unpaired two-tailed *t*-test. ns: not significant; * *p* < 0.05; ** *p* < 0.01; *** *p* < 0.001.

To further validate the impact of ROCK inhibition on the intracellular transport of cholesterol during the process of testosterone synthesis in Leydig cells, we utilized CholEsteryl BODIPY cholesterol fluorescent probe and MitoTracker mitochondrial fluorescent probe to trace free cholesterol and mitochondria in TM3 cells, observing their localization patterns. Fluorescence confocal imaging analysis revealed that 12 h after hCG stimulation, an increased co-localization of MitoTracker and BODIPY fluorescence was evident in the cytoplasm, indicating that a significant amount of cholesterol might shuttle into mitochondria for biochemical metabolism. However, after 1 h of Y-27632 treatment following hCG stimulation, co-localization of MitoTracker and BODIPY in these TM3 cells decreased, and we observed abnormal chunky aggregations of cholesterol and stacking of mitochondria. These changes suggest that the intracellular transport of cholesterol and the spatial dynamics of mitochondria may be influenced by ROCK inhibition. Two hours post-Y-27632 treatment, co-localization of fluorescence probes in TM3 cells was rarely observed. Notably, after 4 and 8 h of Y-27632 treatment, the actin cytoskeleton and cholesterol–mitochondria co-localization showed signs of partial recovery, suggesting a potential compensatory mechanism that may restore intracellular transport dynamics over time (Figure 5A,B). These observations indicate that ROCK-i interferes with the spatial organization of cholesterol and mitochondria in Leydig cells, potentially affecting their coordinated function during steroidogenesis. Additionally, we observed a co-localization pattern of cholesterol and mitochondria along F-actin stress fibers in TM3 cells. This localization pattern was altered in TM3 cells treated with Y-27632 for 2 h. Specifically, we observed that the scattered distribution of Phalloidin, MitoTracker, and BODIPY fluorescence signals in the cytoplasm lost their associative localization, and abnormal aggregations of these three fluorescence signals at cell junctions were noted (Figure 5C,D). This supports a model in which actin filament organization may provide a structural scaffold that facilitates cholesterol trafficking toward mitochondria.

Given the role of ROCK in regulating the expression of genes related to steroid biosynthesis and in cholesterol transport to mitochondria, we hypothesized that ROCK-i could also impact the transcription of certain genes involved in cholesterol transport. The Oxysterol binding protein (OSBP) has been reported to mediate intracellular cholesterol transport through interactions with the Golgi apparatus, playing a significant role in maintaining cholesterol homeostasis [26]. Members of the OSBP-related proteins (ORP) family, including OSBP, ORP4, ORP8, ORP9, and ORP10, have previously been reported to be expressed at relatively high levels in mouse testicular tissue [27] and are believed to be involved in the transport of intracellular oxysterol during the male reproductive process [28]. ABCA1 (ATP Binding Cassette Subfamily A Member 1) is a crucial membrane transporter involved in the transport of cholesterol and phospholipids within cells [29]. Apolipoprotein E (APOE), as a lipoprotein, is involved in the transport and metabolism of cholesterol and is closely associated with the development of cardiovascular diseases [30] and neurodegenerative diseases [31]. Given the significant roles these proteins play in cholesterol transport, we chose genes encoding these proteins as the focus of our study to better understand the regulatory role of ROCK in cholesterol transport in Leydig cells. The qPCR results demonstrated that the expressions of *Osbp*, *Orp4*, and *Orp10* were elevated under hCG stimulation, while their transcription levels were significantly lower in the ROCK-i group compared to the hCG group. Conversely, the expression of *ApoE* was significantly lower in the hCG group compared to the negative control, but it increased under the influence of ROCK-i. *Abca1* expression decreased with hCG stimulation and showed no significant difference in transcription levels between the ROCK-i group and the hCG group (Figure 5E). These findings indicate that in Leydig cells, both hCG signaling and ROCK inhibitors play significant roles in regulating cholesterol transport, which is crucial for testosterone biosynthesis. Specifically, hCG enhances cholesterol transport by regulating the expression of genes such as *Osbp*, *Orp4*, *Orp10*, *Abca1*, and *ApoE*. In contrast, ROCK-i disrupts cholesterol transport by inhibiting the expression of *Osbp*, *Orp4*, and *Orp10*, while promoting the transcription of *ApoE*. These opposing regulatory effects suggest that hCG and ROCK-i modulate steroidogenesis through distinct transcriptional pathways, possibly affecting cholesterol availability for mitochondrial steroid conversion.

In summary, our results support a model in which ROCK contributes to testosterone biosynthesis by coordinating cholesterol transport and mitochondrial dynamics via actin cytoskeleton remodeling. Inhibition of ROCK activity leads to disassembly of actin filaments, disruption of cholesterol–mitochondria co-localization, and altered expression of cholesterol trafficking genes, all of which may collectively reduce testosterone output.

### 3.9. Transcriptome Sequencing Reveals the Effects of ROCK-i on Steroid Biosynthesis Pathways in Leydig Cells

**Figure 6:**
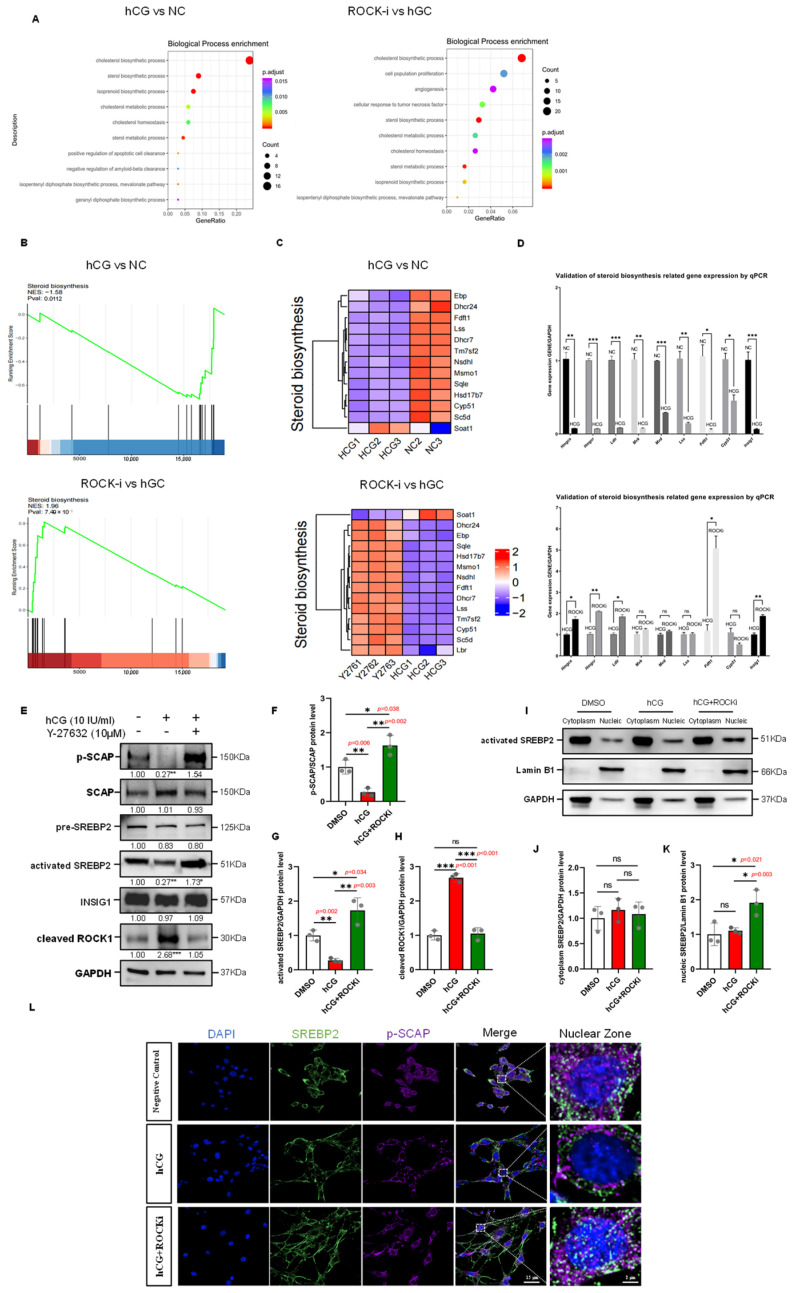
Steroid Biosynthesis regulated by hCG stimulation and ROCK-i in Leydig Cells through SREBP2-Mediated Transcriptional Control. (**A**) GO enrichment bubble chart. Setting *p*-adjust < 0.05 as the threshold and selecting the top 10 pathways, bubble charts were used to visualize these results. The y-axis represents GO terms, the x-axis represents the ratio of genes enriched in each term to the total number of genes, the color indicates the *p*-adjust value, with redder colors indicating greater significance, and the bubble size represents the number of genes enriched in each term, with larger bubbles indicating more genes. (**B**) GSEA enrichment analysis results. The y-axis represents the enrichment score (ES), and the Enrichment Score line graph shows the ES value at each position during calculation. The x-axis represents the rank value distribution of all genes after sorting. In the heatmap, red areas correspond to genes highly expressed in the experimental group, and blue areas correspond to genes with low expression in the experimental group. Lines mark the positions of gene set members in the gene rank list. (**C**) Heatmap analysis of steroid biosynthesis gene set expression levels in the negative control, hCG, and ROCK-i groups. (**D**) qPCR validation of steroid biosynthesis-related genes (*Hmgcs*, *Hmgcr*, *Ldlr*, *Mvk*, *Mvd*, *Lss*, *Fdft1*, *Cyp51*, and *Insig1*) in TM3 cells under the following conditions: NC (Negative Control), HCG (hCG-treated 12 h), and ROCKi (Y-27632-treated 2 h following hCG treatment). Gene expression was normalized to *Gapdh* and presented as mean ± SEM (*n* = 3), with expression in the NC group set to 1. For all these genes, expression levels were significantly downregulated in the hCG group compared to the NC group. In contrast, *Hmgcs*, *Hmgcr*, *Ldlr*, *Fdft1*, and *Insig1* were significantly upregulated in the ROCKi group relative to the hCG group. The expression of *Mvk*, *Mvd*, *Lss*, and *Cyp51* showed no significant difference between the ROCKi and hCG groups. Statistical significance was determined by unpaired two-tailed *t*-test. ns: not significant; * *p* < 0.05; ** *p* < 0.01; *** *p* < 0.001. (**E**) Western Blot analysis of SREBP2 complex components in the hCG and ROCK-i groups. Y-27632 treatment was applied following 12 h of hCG stimulation, with GAPDH used as a loading control. Data are mean of *n* = 3. Values were normalized to the DMSO group; * *p* < 0.05; ** *p* < 0.01; *** *p* < 0.001. (**F**) Quantification of phospho-SCAP/SCAP protein ratio from (**E**), normalized to DMSO (*n* = 3). * *p* < 0.05; ** *p* < 0.01. (**G**) Quantification of results of Western Blot analysis of activated-SREBP2 from (**E**), normalized to DMSO (*n* = 3). * *p* < 0.05; ** *p* < 0.01. (**H**) Quantification of results of Western Blot analysis of cleaved-ROCK1 from (**E**), normalized to DMSO (*n* = 3). ns: not significant; *** *p* < 0.001. (**I**) Western Blot analysis of activated SREBP2 in subcellular fractions in the hCG and ROCK-i groups. Y-27632 treatment was applied following 12 h of hCG stimulation. Lamin B1 was used as a nuclear protein loading control, and GAPDH was used as a cytoplasmic protein loading control. (**J**) Quantification of results of Western Blot analysis of cytoplasm SREBP2 from (**I**), normalized to DMSO (*n* = 3). ns: not significant. (**K**) Quantification of results of Western Blot analysis of nucleic SREBP2 from (**I**), normalized to DMSO (*n* = 3). ns: not significant; * *p* < 0.05. (**L**) Immunofluorescence of cell coverslips shows changes in nuclear expression of SREBP2 in TM3 cells. Y-27632 treatment was applied following 12 h of hCG stimulation. Green fluorescence indicates activated SREBP2; purple fluorescence indicates p-SCAP; nuclei are stained with DAPI (blue). The dashed box areas are magnified. Scale bars: original areas: 15 µm; magnified areas: 2 µm.

To delve deeper into how ROCK-i influences testosterone biosynthesis at the transcriptional level, we performed high-throughput transcriptome sequencing analysis on TM3 cells affected by hCG stimulation and ROCK inhibition. By setting a threshold of a 1.5-fold change and a *p*-value of 0.05, we identified 42 upregulated and 40 downregulated genes in the hCG-treated group. Furthermore, compared to the hCG-treated group, we observed 167 upregulated and 260 downregulated genes in the ROCK-i-treated group (Figure S5A). We employed the R (version 4.3.2) to create heatmaps of the differentially expressed genes, which visually depicted the variations in gene expression patterns and levels among the negative control group, hCG group, and ROCK-i group (Figure S5B). Volcano plot analysis of the differentially expressed genes revealed that several cholesterol biosynthesis-related genes, such as *Lss*, *Mvd*, *Msmo1*, *Sqle*, and *Acat2*, exhibited opposing trends of expression between the hCG and ROCK-i groups (Figure S5C). Using the ClusterProfiler package (version 4.10.0), we analyzed the Gene Ontology (GO) enrichment results and found significant enrichment of pathways related to cholesterol biosynthesis, cholesterol metabolism, cholesterol homeostasis, and steroid biosynthesis in both the hCG and ROCK-i groups (Figure 6A). Additionally, we identified significantly enriched pathways from the Kyoto Encyclopedia of Genes and Genomes (KEGG) database. The KEGG enrichment analysis revealed significant enrichment of the steroid biosynthesis and terpenoid backbone biosynthesis pathways in both groups (Figure S5D). We also conducted Gene Set Enrichment Analysis (GSEA) to assess the distribution trends of the Steroid biosynthesis gene set in the hCG and ROCK-i groups. The results showed a general downregulation of genes in this set in the hCG group with a Normalized Enrichment Score (NES) of -1.58 (hCG group vs NC group); conversely, these genes were generally upregulated in the ROCK-i group, with an NES of 1.96 (ROCK-i group vs hCG group, Figure 6B). A heatmap of the Steroid biosynthesis gene set confirmed the GSEA results, displaying the expression patterns of these genes in the hCG and ROCK-i groups (Figure 6C). Based on these findings, we inferred that exogenous hCG signaling and ROCK inhibition have opposing effects on the transcription levels of steroid biosynthesis-related genes. To validate this inference, we selected a group of significantly differentially expressed steroid biosynthesis-related genes from the transcriptome sequencing results, including *Hmgcs*, *Hmgcr*, *Ldlr*, *Mvk*, *Mvd*, *Lss*, *Fdft1*, *Cyp51*, and *Insig1*, and measured their expression via qPCR. qPCR results indicated that the transcription levels of these 9 genes were significantly lower in the hCG group compared to the negative control group, while the transcription levels of five genes, including *Hmgcs*, *Hmgcr*, *Ldlr*, *Fdft1*, and *Insig1*, were significantly higher in the ROCK-i group compared to the hCG group (Figure 6D). Taken together, these findings indicate that hCG signaling and ROCK inhibition may exert opposing regulatory influences on genes involved in steroid biosynthesis in Leydig cells. The hCG pathway appears to suppress the transcription of key steroidogenic genes, while ROCK-i treatment is associated with transcriptional activation of a subset of these genes, such as *Fdft1* and *Hmgcs*. These results suggest that ROCK activity may contribute to hCG-mediated transcriptional suppression of steroidogenic pathways, and its inhibition could relieve this suppression via an alternate regulatory mechanism.

### 3.10. ROCK-i Modifies the Activation of the Steroid Biosynthesis-Related Transcription Factor SREBP2

Steroid biosynthesis is a complex, energy-consuming process regulated in mammals by intracellular cholesterol levels, ATP content, and hormones (primarily insulin and glucagon). The rate-limiting step in this pathway is the biotransformation reaction catalyzed by HMG-CoA reductase. Our transcriptome sequencing and subsequent qPCR validation in Leydig cells revealed that *Insig1* expression was downregulated by hCG stimulation and elevated following ROCK inhibition (Figure 6D), suggesting potential involvement of the SCAP/SREBP axis.

Sterol regulatory element-binding proteins (SREBPs) are crucial transcription factors that regulate the transcription of several genes related to steroid biosynthesis, including those encoding HMG-CoA reductase (*Hmgcr*) and the low-density lipoprotein receptor (*Ldlr*) [32]. SREBPs are synthesized as precursors bound to the endoplasmic reticulum. When cellular cholesterol and oxysterol levels are high, SREBP forms a complex with SCAP (SREBP cleavage-activating protein) and INSIG (insulin-induced protein) that anchors it to the ER. In contrast, when cholesterol and oxysterol levels drop, SCAP and INSIG, acting as sterol level sensors, respond to this change. INSIG is then ubiquitinated and degraded [33], allowing the SCAP/SREBP complex to migrate to the Golgi apparatus where S1P and S2P, with the aid of SCAP, cleave SREBP, releasing its bHLH regulatory domain to enter the nucleus and bind to DNA at sterol response elements, thus promoting the transcription of cholesterol biosynthesis-related genes [34,35].

The SREBP family in mammals includes SREBP1a, SREBP1c, and SREBP2, each playing distinct roles in gene regulation, with SREBP2 primarily regulating cholesterol synthesis [36]. This reflects their specific functions in different metabolic pathways. To explore the impact of ROCK-i on steroid biosynthesis, based on transcriptome sequencing results, we hypothesized that hCG and ROCK-i influence the transcription of related genes by altering the functional state of SREBP2. Protein immunoblotting of Leydig cells from the hCG and ROCK-i groups showed that with an increase in cleaved ROCK1 expression, the phosphorylation level of SCAP decreased in the hCG group; conversely, with a significant decrease in cleaved ROCK1 expression, the phosphorylation level of SCAP significantly increased in the ROCK-i group (Figure 6E–H). Subcellular fractionation followed by Western Blot revealed that the expression level of activated SREBP2 in the nuclear proteins of the ROCK-i group significantly increased (Figure 6I–K). Immunofluorescence experiments showed that under hCG stimulation, both nuclear SREBP2 and phosphorylated SCAP signals were diminished, whereas ROCK-i treatment enhanced their nuclear localization (Figure 6L).

### 3.11. Selective Silencing of ROCK1 and ROCK2 Reveals Their Isoform-Specific Roles in Cytoskeletal Remodeling in TM3 Cells

**Figure 7:**
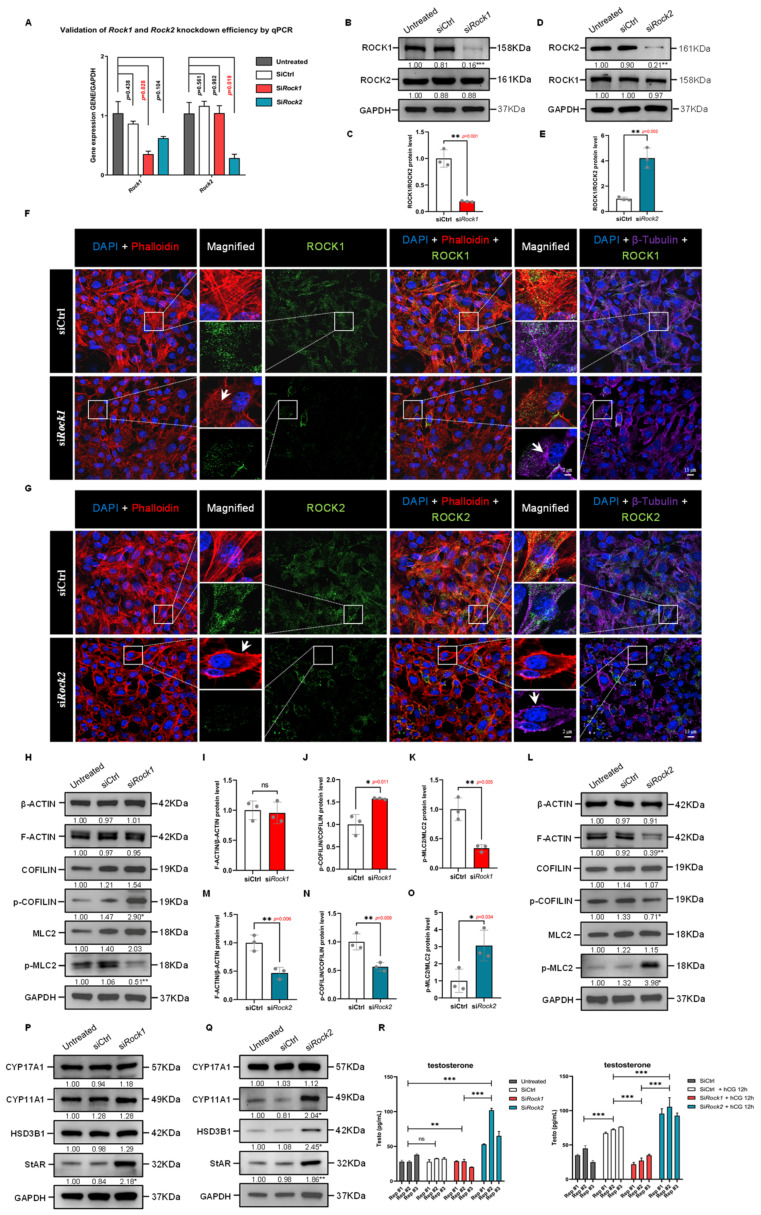
Differential effects of ROCK1 and ROCK2 knockdown on cytoskeleton organization and steroidogenesis in TM3 cells. (**A**) qPCR analysis of *Rock1* and *Rock2* mRNA in TM3 cells treated with siCtrl, si*Rock1*, or si*Rock2*. Expression was normalized to *Gapdh* and the Untreated group (*n* = 3). *Rock1* expression was significantly reduced by si*Rock1* (*p* = 0.028), while *Rock2* was significantly reduced by si*Rock2* (*p* = 0.019). *p* values by unpaired *t*-test. (**B**) Western Blot analysis of ROCK1 and ROCK2 in TM3 cells transfected with siRock1. GAPDH was used as loading control. ROCK1 levels were significantly reduced in si*Rock1* cells compared to siCtrl (*p* < 0.001); ROCK2 levels were unchanged. Data are mean of *n* = 3. Values were normalized to the Untreated group. *** *p* < 0.001. (**C**) Quantification of ROCK1/ROCK2 protein ratio from (**B**), normalized to siCtrl (*n* = 3). The ratio was significantly reduced in si*Rock1* cells (*p* = 0.001), indicating specific knockdown of ROCK1 without affecting ROCK2. ** *p* < 0.01. (**D**) Western Blot analysis of ROCK1 and ROCK2 in TM3 cells transfected with si*Rock2*. GAPDH served as loading control. ROCK2 levels were significantly reduced in si*Rock2* cells compared to siCtrl (*p* < 0.01); ROCK1 levels remained unchanged (*n* = 3). Values were normalized to the Untreated group. ** *p* < 0.01. (**E**) Quantification of ROCK1/ROCK2 protein ratio from (**D**), normalized to siCtrl (*n* = 3). The ratio was significantly increased in si*Rock2* cells (*p* = 0.002), suggesting effective ROCK2 knockdown with no apparent change in ROCK1 levels. ** *p* < 0.01. (**F**) Immunofluorescence staining of TM3 cells showing DAPI (blue), F-actin (Phalloidin, red), ROCK1 (green), and β-Tubulin (purple) in siCtrl and si*Rock1* groups. In siCtrl cells, ROCK1 signal was broadly distributed along both actin filaments and microtubules, with organized cytoskeletal structures. si*Rock1* cells showed reduced ROCK1 signal, particularly in cytoskeletal regions, accompanied by disrupted actin stress fibers and aberrant microtubule aggregation as arrows indicated. The dashed box areas are magnified. Scale bars: original areas: 20 μm; magnified areas: 5 μm. (**G**) Immunofluorescence staining of TM3 cells showing DAPI (blue), F-actin (Phalloidin, red), ROCK2 (green), and β-Tubulin (purple) in siCtrl and si*Rock2* groups. In siCtrl cells, ROCK2 colocalized with organized actin and microtubule networks. si*Rock2* cells exhibited reduced ROCK2 signal and distinct cytoskeletal alterations, including cortical F-actin formation and clustered microtubules as arrows indicated. The dashed box areas are magnified. Scale bars: original areas: 20 μm; magnified areas: 5 μm. (**H**) Western Blot analysis of β-ACTIN, F-ACTIN, COFILIN, phospho-COFILIN, MLC2, and phospho-MLC2 in TM3 cells treated with si*Rock1* (*n* = 3). Expression was normalized to GAPDH. No significant changes were observed in total β-ACTIN, F-ACTIN, COFILIN, or MLC2 levels. Phospho-COFILIN levels were increased (*p* < 0.05), while phospho-MLC2 levels were decreased (*p* < 0.01) in the si*Rock1* group compared to siCtrl. Values were normalized to the Untreated group. * *p* < 0.05; ** *p* < 0.01. (**I**) Quantification of the F-ACTIN/β-ACTIN protein ratio from (**H**), normalized to siCtrl (*n* = 3). No significant difference was observed between si*Rock1* and siCtrl groups. ns: not significant; *** *p* < 0.001. (**J**) Quantification of phospho-COFILIN/COFILIN protein ratio from (**H**), normalized to siCtrl (*n* = 3). The ratio was significantly increased in si*Rock1* cells compared to siCtrl (*p* = 0.011). * *p* < 0.05. (**K**) Quantification of phospho-MLC2/MLC2 protein ratio from (**H**), normalized to siCtrl (*n* = 3). The ratio was significantly decreased in si*Rock1* cells compared to siCtrl (*p* = 0.005). ** *p* < 0.01. (**L**) Western Blot analysis of β-ACTIN, F-ACTIN, COFILIN, phospho-COFILIN, MLC2, and phospho-MLC2 in TM3 cells following ROCK2 knockdown (*n* = 3). Expression was normalized to GAPDH. No significant changes were observed in β-ACTIN, COFILIN, or MLC2 levels. F-ACTIN expression was reduced in si*Rock2* cells compared to siCtrl (*p* < 0.01), accompanied by decreased phospho-COFILIN (*p* < 0.05) and increased phospho-MLC2 levels (*p* < 0.05). Values were normalized to the Untreated group. * *p* < 0.05; ** *p* < 0.01. (**M**) Quantification of the F-ACTIN/β-ACTIN protein ratio from (**L**), normalized to siCtrl (*n* = 3). The ratio was significantly decreased in si*Rock2* cells compared to siCtrl (*p* = 0.006). ** *p* < 0.01. (**N**) Quantification of phospho-COFILIN/COFILIN protein ratio from (**L**), normalized to siCtrl (*n* = 3). The ratio was significantly decreased in si*Rock2* cells compared to siCtrl (*p* = 0.009). ** *p* < 0.01. (**O**) Quantification of phospho-MLC2/MLC2 protein ratio from (**L**), normalized to siCtrl (*n* = 3). The ratio was significantly increased in si*Rock2* cells compared to siCtrl (*p* = 0.034). * *p* < 0.05. (**P**) Western Blot analysis of steroidogenic enzymes in TM3 cells following ROCK1 knockdown (*n* = 3). Expression was normalized to GAPDH. No significant changes were observed in CYP17A1, CYP11A1, or HSD3B1 expression. StAR protein levels were significantly increased in si*Rock1* cells compared to siCtrl (*p* < 0.05). Values were normalized to the Untreated group. * *p* < 0.05. (**Q**) Western Blot analysis of steroidogenic enzymes in TM3 cells following ROCK2 knockdown. Mean protein levels of CYP11A1, HSD3B1, and StAR were significantly increased in the si*Rock2* group compared to siCtrl (*p* < 0.05, *p* < 0.01 for StAR), while CYP17A1 showed no significant change (*n* = 3). GAPDH was used as loading control, and values were normalized to the Untreated group. * *p* < 0.05; ** *p* < 0.01. (**R**) ELISA quantification of testosterone levels in TM3 cells following ROCK1 or ROCK2 knockdown, with or without hCG stimulation. Data are presented as mean ± SEM (pg/mL; *n* = 3). Without hCG treatment, no significant difference was observed between Untreated (31.30 ± 3.35) and siCtrl (30.83 ± 1.52) groups. Testosterone levels were significantly reduced in si*Rock1* (25.73 ± 2.77, *p* < 0.01) and increased in si*Rock2* (73.37 ± 14.74, *p* < 0.001) compared to Untreated. si*Rock2* levels were also significantly higher than si*Rock1* (*p* < 0.001). With hCG treatment (12 h, 10 IU/mL), testosterone levels increased from 34.93 ± 5.75 (siCtrl) to 72.03 ± 2.72 (siCtrl + hCG, *p* < 0.001). This hCG-induced increase was significantly suppressed in si*Rock1* + hCG cells (28.00 ± 3.77, *p* < 0.001 vs. siCtrl + hCG), and further enhanced in si*Rock2* + hCG cells (97.93 ± 3.93, *p* < 0.001 vs. siCtrl + hCG). si*Rock2* + hCG levels were significantly higher than si*Rock1* + hCG (*p* < 0.001). ns: not significant; ** *p* < 0.01; *** *p* < 0.001.

To delineate the isoform-specific roles of ROCK1 and ROCK2 in Leydig cell cytoskeletal organization, we employed siRNA-mediated knockdown of *Rock1* and *Rock2* in TM3 cells. qPCR and immunoblotting confirmed efficient silencing of each target without off-target suppression (Figure 7A–E). Immunofluorescence analysis showed that ROCK1 deficiency led to loss of F-actin bundles and induced microtubule disorganization, while ROCK2 knockdown promoted enhanced cortical F-actin ring formation and microtubule aggregation (Figure 7F,G). These results suggest divergent roles for ROCK1 and ROCK2 in actin-microtubule network maintenance.

Western Blot analysis of F-actin regulatory proteins revealed opposing changes in the phosphorylation states of COFILIN and MLC2. In ROCK1-deficient cells, increased p-COFILIN and decreased p-MLC2 levels were observed, indicative of reduced actomyosin contractility and stabilization of actin filaments (Figure 7H–K). These findings align with prior reports showing that ROCK1 regulates central stress fiber formation through MLC2-mediated myosin activation [37,38]. In contrast, ROCK2 knockdown decreased p-COFILIN and increased p-MLC2, along with reduced F-ACTIN/β-ACTIN ratios (Figure 7L–O), suggesting enhanced actin turnover and elevated contractile tension. Similar ROCK2-specific roles in cytoskeletal destabilization and contractility reinforcement have been described in other cell types [38,39]. Taken together, these data support that ROCK1 primarily supports cytoskeletal organization via stabilization of actin structures, whereas ROCK2 may act to restrain excessive actin turnover and myosin activation.

### 3.12. ROCK1 and ROCK2 Oppositely Control Testosterone Synthesis

Given the critical role of cytoskeletal dynamics in cholesterol trafficking and steroidogenesis, we next evaluated the impact of ROCK1 and ROCK2 knockdown on steroidogenic gene expression and testosterone production. In ROCK1-deficient cells, levels of CYP17A1, CYP11A1, and HSD3B1 remained unchanged, while StAR expression showed a modest but significant increase (Figure 7P). As StAR functions to facilitate cholesterol transfer to the inner mitochondrial membrane, its upregulation may reflect a compensatory response to impaired cytoskeletal support for mitochondrial cholesterol transport. In contrast, ROCK2 knockdown markedly upregulated CYP11A1, HSD3B1, and StAR protein levels (Figure 7Q), consistent with enhanced steroidogenic activity. These isoform-specific effects underscore functional divergence in how ROCK1 and ROCK2 interface with steroidogenic machinery.

Consistent with these molecular shifts, ELISA measurements showed that testosterone levels decreased significantly in si*Rock1* cells (Figure 7R, left), supporting an essential role of ROCK1 in basal steroid biosynthesis. In contrast, si*Rock2* knockdown led to a marked increase in testosterone secretion, more than doubling that of the control. (Figure 7R). Notably, upon hCG stimulation, testosterone output was enhanced in control cells as expected, but severely blunted in the si*Rock1* group and significantly upregulated in the si*Rock2* group. These results suggest that ROCK1 is necessary for the hormonal induction of testosterone synthesis, whereas ROCK2 suppression unmasks a heightened steroidogenic capacity even under hCG stimulus. Collectively, the opposing effects on steroidogenic output imply that ROCK1 is essential for coordinating cytoskeleton-assisted cholesterol trafficking during testosterone synthesis, while ROCK2 negatively regulates the steroidogenic machinery, possibly by restricting actomyosin activity.

### 3.13. ROCK1 and ROCK2 Exert Opposing Effects on Cytoskeletal Architecture and Cholesterol–Mitochondria Coupling

**Figure 8:**
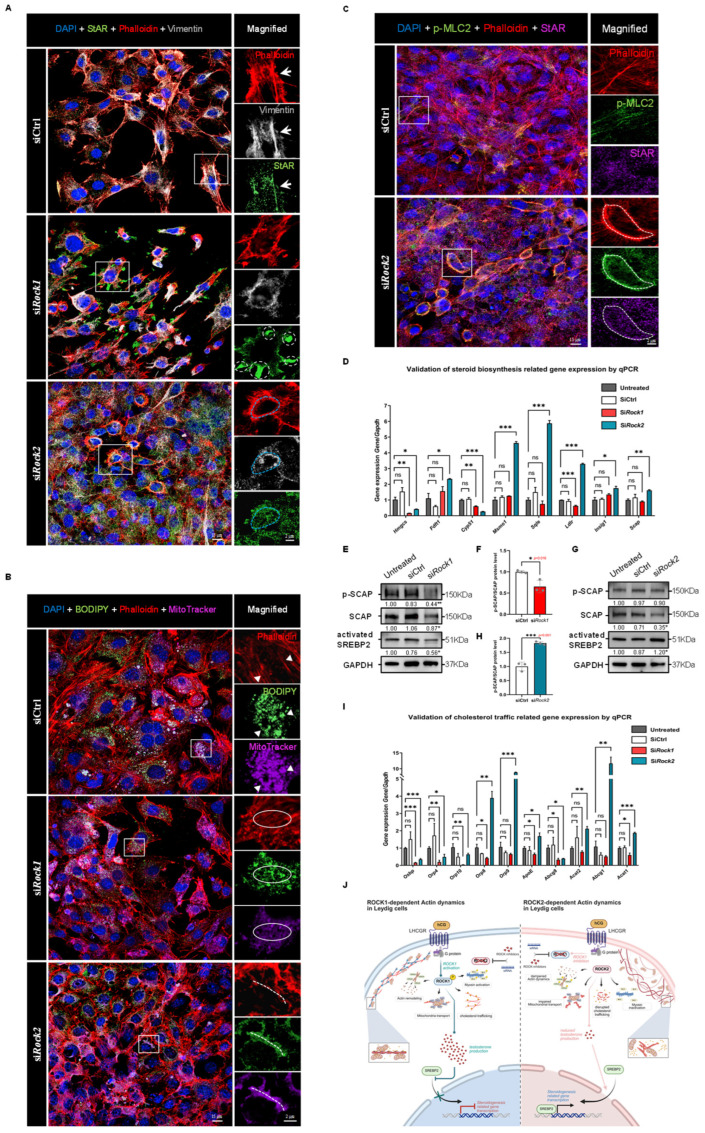
Knockdown of ROCK1 and ROCK2 reveals opposing effects on cytoskeleton-dependent cholesterol trafficking and SREBP2-driven steroidogenic gene expression. (**A**) Immunofluorescence staining of TM3 cells showing DAPI (blue), F-actin (Phalloidin, red), StAR (green), and Vimentin (gray) in siCtrl, si*Rock1*, and si*Rock2* groups. In siCtrl cells, StAR signal was distributed along cytoskeletal regions marked by F-actin and Vimentin (arrows). In si*Rock1* cells, StAR appeared mislocalized and clustered near cell–cell junctions, with limited colocalization with cytoskeletal structures (white circles). In contrast, si*Rock2* cells showed enhanced colocalization of StAR with Vimentin and cortical F-actin (blue circles). The box areas are magnified. Scale bars: original areas: 15 μm; magnified areas: 2 μm. (**B**) Immunofluorescence staining of TM3 cells showing DAPI (blue), F-actin (Phalloidin, red), cholesterol (BODIPY, green), and mitochondria (MitoTracker, purple) in siCtrl, si*Rock1*, and si*Rock2* groups. In siCtrl cells, BODIPY and MitoTracker signals were colocalized along stress fiber regions marked by F-actin (triangles). In si*Rock1* cells, disrupted F-actin structures were associated with loss of BODIPY–MitoTracker colocalization (circles). In si*Rock2* cells, cortical F-actin rings showed enhanced colocalization with both cholesterol and mitochondria (dotted lines). The box areas are magnified. Scale bars: original areas: 15 μm; magnified areas: 2 μm. (**C**) Immunofluorescence staining of TM3 cells showing DAPI (blue), F-actin (Phalloidin, red), phospho-MLC2 (green), and StAR (purple) in siCtrl and si*Rock2* groups. In siCtrl cells, p-MLC2 signal was weak and did not appreciably overlap with StAR staining. In si*Rock2* cells, p-MLC2 was enriched in cortical F-actin regions and colocalized with StAR signals (circles), suggesting spatial coupling between activated MLC2 and mitochondrial cholesterol transport. The box areas are magnified. Scale bars: original areas: 15 μm; magnified areas: 2 μm. (**D**) qPCR analysis of cholesterol biosynthesis-related genes in TM3 cells following ROCK1 or ROCK2 knockdown. Gene expression was normalized to *Gapdh* and the Untreated group (set as 1; *n* = 3). No significant differences were observed between Untreated and siCtrl groups. *Hmgcs* and *Cyp51* expression was significantly decreased in both si*Rock1* and si*Rock2* groups. *Fdft1*, *Msmo1*, *Sqle*, *Ldlr*, *Insig1*, and *Scap* were all significantly upregulated in si*Rock2* cells, while their expression remained unchanged or decreased in si*Rock1* cells. These results suggest distinct effects of ROCK1 and ROCK2 knockdown on transcriptional regulation of cholesterol biosynthesis pathways. ns: not significant; * *p* < 0.05; ** *p* < 0.01; *** *p* < 0.001. (**E**) Western Blot analysis of SCAP, phospho-SCAP (p-SCAP), and activated SREBP2 in TM3 cells following ROCK1 knockdown (*n* = 3). GAPDH served as loading control, and signal intensities were normalized to the Untreated group (set as 1). SCAP, p-SCAP, and activated SREBP2 levels were significantly decreased in si*Rock1* cells compared to siCtrl (*p* < 0.05, *p* < 0.01, and *p* < 0.05, respectively). * *p* < 0.05; ** *p* < 0.01. (**F**) Quantification of phospho-SCAP/SCAP protein ratio from (**E**), normalized to siCtrl (*n* = 3). The ratio was significantly decreased in si*Rock1* cells compared to siCtrl (*p* = 0.016). * *p* < 0.05. (**G**) Western Blot analysis of SCAP, phospho-SCAP (p-SCAP), and activated SREBP2 in TM3 cells following ROCK2 knockdown (*n* = 3). GAPDH was used as loading control, and signal intensities were normalized to the Untreated group (set as 1). While SCAP levels were slightly decreased and p-SCAP levels remained unchanged, activated SREBP2 was significantly increased in si*Rock2* cells compared to siCtrl (*p* < 0.05). * *p* < 0.05. (**H**) Quantification of phospho-SCAP/SCAP protein ratio from (**G**), normalized to siCtrl (*n* = 3). The ratio was significantly increased in si*Rock2* cells compared to siCtrl (*p* < 0.001). *** *p* < 0.001. (**I**) qPCR analysis was performed to assess the mRNA levels of genes involved in cholesterol transport, including *Osbp*, *Orp4*, *Orp10*, *Orp8*, *Orp9*, *ApoE*, *Abcg8*, *Acat2*, *Abcg1*, and *Acat1*, across four groups: Untreated, SiCtrl, Si*Rock1*, and Si*Rock2*. Gene expression was normalized to *Gapdh* and presented as mean ± SEM (*n* = 3), with expression in the Untreated group set to 1. No significant differences were observed between Untreated and SiCtrl groups. *Osbp* and *Orp4* were significantly downregulated in both Si*Rock1* and Si*Rock2* groups. In the Si*Rock1* group, expression of *Orp10*, *Orp8*, *ApoE*, and *Acat1* was significantly decreased compared to Untreated, whereas these genes were either unchanged or significantly upregulated in the Si*Rock2* group. Notably, *Orp8*, *Orp9*, *ApoE*, *Acat2*, *Abcg1*, and *Acat1* were significantly upregulated in the Si*Rock2* group. These changes suggest distinct regulatory roles of ROCK1 and ROCK2 in cholesterol mobilization. Statistical significance was determined by unpaired two-tailed *t*-test. ns: not significant; * *p* < 0.05; ** *p* < 0.01; *** *p* < 0.001. (**J**) Proposed model illustrating the distinct roles of ROCK1 and ROCK2 in regulating testosterone biosynthesis via cytoskeletal remodeling. ROCK1 promotes cytoskeletal support for cholesterol trafficking and enhances SCAP phosphorylation and SREBP2 activation, thereby facilitating steroidogenic gene expression. However, excessive testosterone production triggered by ROCK2 knockdown feeds back to suppress SCAP-SREBP2 signaling. In contrast, ROCK2 normally restrains actomyosin dynamics and SCAP-SREBP2 activation; its knockdown relieves this suppression, boosting both cholesterol transport and steroidogenic gene expression. These findings highlight a dual regulatory mechanism whereby ROCK1 and ROCK2 exert opposing influences on testosterone synthesis and its feedback control of cholesterol biosynthesis. Created in BioRender. Xu, K. (2025) https://BioRender.com/mwrghma (accessed on 30 July 2025).

To investigate the cytoskeletal basis underlying these opposing steroidogenic outcomes, we examined their roles in cytoskeleton-dependent intracellular trafficking. Immunofluorescence staining of TM3 cells revealed that in control cells, StAR localized along cytoskeletal tracks demarcated by F-actin and Vimentin, suggesting efficient targeting to mitochondria (Figure 8A). ROCK1 knockdown led to disorganization of F-actin stress fibers and Vimentin collapse, with StAR aberrantly accumulating at intercellular junctions, spatially dissociated from the cytoskeletal scaffold (Figure 8A). In contrast, ROCK2-deficient cells exhibited pronounced cortical F-actin structures where StAR, Vimentin, and MitoTracker signals strongly overlapped, implying enhanced spatial coupling between cholesterol trafficking machinery and mitochondria (Figure 8A).

To directly assess mitochondrial cholesterol delivery, we co-labeled cholesterol (BODIPY) and mitochondria (MitoTracker). In control cells, cholesterol–mitochondria colocalization was tightly aligned with linear F-actin bundles (Figure 8B). This spatial alignment was severely disrupted upon ROCK1 silencing, consistent with cytoskeletal disintegration and loss of directional transport. Conversely, ROCK2 knockdown induced cortical F-actin rings that closely aligned with mitochondrial cholesterol accumulation, indicating enhanced transport efficiency. Notably, phospho-MLC2, a marker of actomyosin activation, was barely detectable in control cells but markedly upregulated in ROCK2-deficient cells, with strong colocalization with both StAR and cortical actin (Figure 8C). This suggests that ROCK2 knockdown induces a compensatory increase in myosin II-driven contractility, which, when coupled with actin reorganization, may facilitate peripherally directed mitochondrial docking and cholesterol influx. These cytoskeletal rearrangements likely enhance the spatial coupling between cholesterol, mitochondria, and the steroidogenic machinery, providing a physical basis for increased testosterone synthesis following ROCK2 depletion.

### 3.14. ROCK Isoforms Differentially Regulate the SCAP–SREBP2 Axis in Response to Testosterone Feedback

We next evaluated whether ROCK1 and ROCK2 differentially modulate transcriptional programs governing cholesterol biosynthesis and transport. ROCK1 knockdown led to broad suppression of genes involved in de novo cholesterol biosynthesis, including *Hmgcs*, *Cyp51*, and *Ldlr* (Figure 8D), indicating reduced lipid supply. In contrast, ROCK2 knockdown resulted in selective upregulation of genes associated with sterol synthesis (e.g., Fdft1, *Msmo1*, *Sqle*) and cholesterol trafficking (e.g., Orp8, *Orp9*, *ApoE*, *Abcg1*, *Acat1*) (Figure 8I), suggesting enhanced lipid mobilization. These opposing transcriptional patterns suggest that ROCK1 and ROCK2 engage distinct feedback loops.

To investigate whether these transcriptional effects are mediated via SREBP2, the master regulator of cholesterol metabolism, we analyzed components of the SCAP–SREBP2 complex. ROCK1 knockdown markedly decreased total SCAP and its phosphorylated form (Figure 8E), as well as nuclear (active) SREBP2 (Figure 8F), suggesting suppressed SREBP2 processing. This is consistent with reduced steroid output following ROCK1 depletion. Conversely, ROCK2 knockdown resulted in increased nuclear SREBP2 accumulation (Figure 8G), despite only modest changes in total SCAP and a paradoxical increase in p-SCAP/SCAP ratio (Figure 8H). This upregulation of SREBP2 is likely secondary to the marked increase in testosterone secretion (Figure 7R), triggering a homeostatic feedback aimed at restraining excessive cholesterol influx. Our findings support a model in which ROCK1 facilitates SCAP phosphorylation and SREBP2 maturation under basal conditions, sustaining steroidogenic capacity. However, testosterone overproduction following ROCK2 knockdown induces SREBP2-mediated cholesterol uptake and biosynthesis, possibly as part of an intrinsic buffering mechanism. Thus, ROCK1 and ROCK2 function as reciprocal modulators of steroidogenesis through distinct effects on cytoskeleton-mediated cholesterol delivery and transcriptional control of lipid metabolism (Figure 8J).

## 4. Discussion

The synthesis and secretion of steroid hormones are complex, with multistep processes involving precursor mobilization, enzymatic conversion, and inter-organelle substrate trafficking. Among these steps, the translocation of cholesterol to the inner mitochondrial membrane is considered the rate-limiting step in testosterone biosynthesis. Cholesterol can be sourced endogenously via de novo biosynthesis, released from membranes or lipid droplets, or acquired exogenously through endocytosis or active uptake, and must be delivered through the ER-mitochondria interface with the assistance of StAR protein [5,15]. Cytoskeletal systems—including microtubules, intermediate filaments, and microfilaments—are thought to play central roles in this intracellular transport network.

Previous studies have implicated various cytoskeletal elements in steroid hormone production. Lipid droplets, essential reservoirs of cholesterol in steroidogenic cells, rely on cytoskeletal structures to coordinate their spatial distribution and interactions with mitochondria [40]. Intermediate filaments provide structural support [41], while actomyosin contraction and cAMP-mediated phosphorylation events remodel actin networks and regulate transport fidelity [41,42]. Disruption of Vimentin or pharmacological destabilization of microtubules has been shown to impair steroid biosynthesis [43,44,45], while cAMP-dependent phosphorylation of Vimentin induces conformational changes in the specialized actin-myosin structure “actomyosin ring” [46]. In summary, the cytoskeleton acts as a platform for intracellular traffic in the process of steroidogenesis, although findings remain context-dependent across species and cell types.

In the present study, we investigated the role of Rho-associated protein kinases—ROCK1 and ROCK2—in regulating cytoskeletal remodeling and its impact on testosterone biosynthesis in murine Leydig cells. Using transcriptomics and immunostaining, we confirmed ROCK1/2 expression is enriched in Leydig cells. Pharmacological inhibition using Y-27632 effectively suppressed ROCK activity within an effective timeframe of 15 min to 4 h, altering actin, microtubule, and intermediate filament structures. This was accompanied by reduced phosphorylation of LIMK, COFILIN, and MLC2, which are key regulators of actin polymerization and myosin activity. Importantly, Y-27632 exhibited no cytotoxicity over 24 h and promoted TM3 cell proliferation while attenuating apoptosis.

We further observed that Y-27632 disrupted the subcellular localization of the steroidogenic enzyme 3β-HSD and interfered with the formation of mitochondrial-targeted cholesterol pools, as visualized by BODIPY and MitoTracker colocalization. These cytoskeletal disruptions impaired StAR-mediated cholesterol trafficking and suppressed testosterone production, as confirmed by ELISA. Transcriptome analysis revealed opposing effects of ROCK inhibition and hCG stimulation on the expression of steroid biosynthesis-related genes, including *Fdft1* and *Hmgcs*, as well as transport regulators *Osbp*, *Orp4*, and *Orp10*.

Notably, the observations from time-course experiments (shown in Figure 5A) revealed a time-dependent compensatory response in TM3 cells following ROCK inhibition. Within 1–2 h of Y-27632 treatment, F-actin disassembly and disrupted cholesterol–mitochondria co-localization were evident, coinciding with suppressed testosterone secretion. However, at 4 and 8 h, we observed partial reassembly of actin filaments and gradual restoration of cholesterol transport to mitochondria, suggesting that Leydig cells may initiate an intrinsic compensatory program to counteract the cytoskeletal and metabolic disruptions caused by ROCK inhibition. This compensation may involve the activation of alternative cytoskeletal regulatory pathways or feedback activation of cholesterol biosynthesis genes, as partially supported by the increased SREBP2 nuclear localization and upregulation of cholesterol synthesis-related genes (e.g., Hmgcs, *Fdft1*) under ROCK inhibition. These findings not only underscore the plasticity of cytoskeletal dynamics in steroidogenic cells but also highlight a temporal limitation in the efficacy of transient ROCK inhibition as a potential therapeutic strategy.

To dissect the functional relevance of ROCK1 and ROCK2 in regulating cytoskeletal architecture in Leydig cells, we employed both pharmacological inhibition using Y-27632 and isoform-specific siRNA knockdown approaches. Y-27632, a widely used pan-ROCK inhibitor, rapidly disrupted actin stress fiber formation, disassembled intermediate filaments, and led to microtubule aggregation within hours of treatment. These acute changes reflected its broad inhibitory effects on both ROCK1 and ROCK2, resulting in pronounced alterations of cytoskeletal dynamics. In contrast, siRNA-mediated knockdown of ROCK1 or ROCK2 offered a more gradual and isoform-specific regulatory landscape. Notably, ROCK1 depletion mimicked Y-27632-induced cytoskeletal disassembly, including reduced F-actin integrity and redistribution of StAR protein, while ROCK2 knockdown induced a distinct increase in cortical F-actin enrichment, suggestive of compensatory remodeling and functional divergence.

These findings highlight the complementary nature of pharmacological and genetic tools: while inhibitors offer temporal control and broad-spectrum inhibition, siRNA approaches provide mechanistic specificity that allows dissection of isoform-dependent effects. The combined application of both strategies not only validates the role of ROCK signaling in cytoskeletal remodeling but also uncovers functional nuances between ROCK1 and ROCK2, which could otherwise be masked by the nonspecificity of Y-27632.

Members of the Rho GTPase family are key regulators of the actin cytoskeleton. ROCK1 and ROCK2, as critical downstream effectors, promote actomyosin contraction by phosphorylating MLC and inactivating myosin phosphatase, while also modulating actin polymerization Via the LIMK-COFILIN axis [7]. Additionally, ROCK phosphorylates intermediate filament proteins like Vimentin, and can influence microtubule stability through phosphorylation of MAP2/Tau and Doublecortin [47,48]. These broad cytoskeletal roles place ROCK signaling at the nexus of structural remodeling and intracellular transport.

Transcriptomic and protein analyses further revealed that hCG-LHCGR signaling activates ROCK1, inducing phosphorylation of LIMK, COFILIN, and MLC2, and reorganizing F-actin to facilitate mitochondrial cholesterol trafficking. Interestingly, this signal also suppresses the transcriptional activity of the SCAP/SREBP2 pathway, a master regulator of endogenous cholesterol synthesis. Conversely, ROCK inhibition reactivates this pathway, increasing SREBP2 nuclear translocation and downstream gene expression. Western Blot confirmed these patterns, showing reciprocal expression of nuclear SREBP2 and phosphorylated SCAP in hCG- and ROCK-i-treated cells.

The identification of distinct and even opposing roles for ROCK1 and ROCK2 in regulating cholesterol transport and steroid biosynthesis has substantial therapeutic implications. In particular, ROCK2 knockdown enhanced testosterone production, accompanied by increased mitochondrial targeting of cholesterol and upregulation of SREBP2-dependent biosynthetic pathways. These findings suggest that ROCK2 may function as a negative regulator of androgen biosynthesis, positioning it as a promising therapeutic target in male hypogonadism and testosterone deficiency syndromes. Belumosudil, an FDA-approved ROCK2 inhibitor, has demonstrated safety and efficacy in fibrotic and inflammatory diseases [49], and our data raise the possibility of repurposing such compounds in andrological contexts, especially for hypogonadal men with impaired Leydig cell function.

Notably, ROCK2-deficient Leydig cells exhibited a striking redistribution of F-actin toward the cell periphery, forming a prominent cortical actin network. This cytoskeletal reorganization, not observed in control or ROCK1-deficient cells, points to a ROCK2-specific role in restraining cortical actin assembly, with potential implications for cholesterol trafficking efficiency. This cortical actin enrichment was spatiotemporally associated with enhanced mitochondrial targeting of cholesterol and increased testosterone production, suggesting that cortical F-actin may serve as a structural scaffold to facilitate directional cholesterol trafficking toward mitochondria. This mechanism provides a new conceptual framework linking actin cytoskeleton polarity to steroidogenic efficiency, with potential parallels to vesicle transport and cell polarity processes in other secretory cell types.

Given that ROCK2 knockdown promotes cortical F-actin accumulation and testosterone output, while ROCK1 knockdown disrupts cytoskeletal integrity and impairs steroidogenesis, we propose a model in which ROCK1 and ROCK2 exert opposing influences on F-actin architecture, thus fine-tuning the balance between structural support and dynamic remodeling required for efficient steroid biosynthesis. This insight not only enhances our understanding of cytoskeletal control of steroidogenesis but also identifies cortical actin polymerization as a key regulatory node in Leydig cell function.

Although ROCK1 and ROCK2 are highly homologous, their substrates and functions in various biological processes differ. However, most ROCK inhibitors lack subtype specificity, which presents technical challenges in studies of subtype-specific functions of ROCK and remains a significant obstacle to translational application [8]. High-throughput drug screening and rational inhibitor design focused on ROCK1/2-selective modulation are urgently needed. Moreover, pharmaceutically inhibitory effect of ROCK2 on testosterone biosynthesis may be transient, and sustained inhibition or combination therapies may be necessary to achieve a prolonged physiological impact. Notably, recent research by Kai et al. has demonstrated that AAV8 vectors can selectively target Leydig cells and restore androgen production in *Lhcgr*-deficient models [50], suggesting that cell-specific ROCK modulation may have clinical potential. Ultimately, understanding the cytoskeletal basis of cholesterol transport may reveal novel targets for treating male reproductive endocrine disorders.

## 5. Conclusions

This study uncovers the isoform-specific regulatory roles of the cytoskeletal modulator ROCK in Leydig cells, highlighting its critical function in coordinating cytoskeletal remodeling and testosterone biosynthesis. We demonstrate that ROCK reshapes the actin cytoskeleton through the phosphorylation of LIMK, COFILIN, and MLC2, thereby coordinating cholesterol transport and enhancing testosterone synthesis in Leydig cells. The findings suggest that hCG-LHCGR signaling activates ROCK1, which promotes F-actin stress fiber formation, facilitating cholesterol transport toward mitochondria. siRNA-mediated knockdown of ROCK1 impairs this process, leading to decreased testosterone synthesis, suppressed expression of steroidogenic genes, and reduced cholesterol availability. In contrast, ROCK2 knockdown induces cortical F-actin remodeling and enhances MLC2 activation, thereby promoting mitochondrial cholesterol targeting and testosterone secretion. This overproduction triggers compensatory feedback that activates the SCAP–SREBP2 axis, stimulating endogenous cholesterol biosynthesis. Together, these findings delineate a distinct functional dichotomy between ROCK1 and ROCK2 in regulating cholesterol trafficking and testosterone output, and provide mechanistic insights for targeting ROCK signaling in the development of male reproductive endocrine therapies.

## Data Availability

The original contributions presented in the study are included in the article/Supplementary Material, further inquiries can be directed to the corresponding authors.

## Supplementary Materials

The following supporting information can be downloaded at: https://www.mdpi.com/article/10.3390/cells14231868/s1, Figure S1: Analyzing of *ROCK* Expression in Human Testicular cells.; Figure S2: The time-dependent effects of ROCK inhibitor on the expression of F-actin remodeling factors in TM3 cells.; Figure S3: Y-27632 Shows No Significant Proliferative Toxicity in TM3 Cells During Its Effective Duration.; Figure S4: Detection of Testosterone in TM3 Cell Culture Medium.; Figure S5: Differential Gene Expression and Enrichment Analysis in TM3 Cells Under hCG and ROCK-i Treatment.; Table S1: Primary Antibodies Information.; Table S2: qPCR Primer Sequences.; Original images of Western Blot.
