# Supplementary material for: Deciphering the Contribution of ROCK-Dependent Actin Cytoskeleton Remodeling to Testosterone Production in Mouse Leydig Cells
Source: Cells. 2025 Nov 26;14(23):1868. doi: 10.3390/cells14231868 (PMC12691457; doi:10.3390/cells14231868)
Supplement: Supplementary file 1 [file cells-14-01868-s001.zip › cells-3074768-supplementary.pdf]

Supplementary Information for

Deciphering the Contribution of ROCK-Dependent Actin Cytoskeleton Remodeling to Testosterone Production in Mouse Leydig Cells

Cells

This file includes:

Figure S1 to S5
Table S1
Table S2
Original images of Western blotting

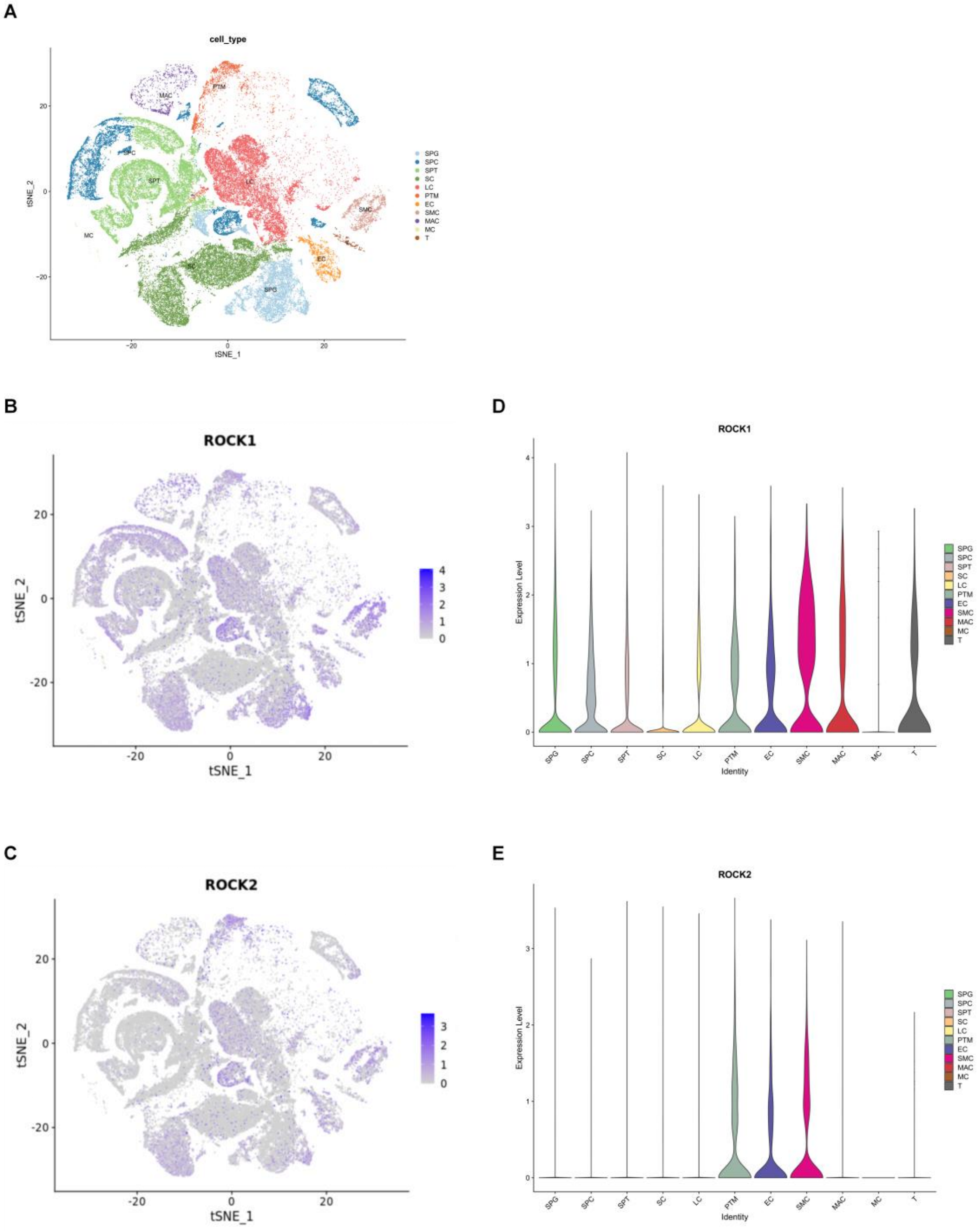

Figure S1. Analyzing of *ROCK* Expression in Human Testicular cells.

T-Distributed Stochastic Neighbor Embedding (t-SNE) plots (A-C) and violin plots (D-E) showing the expression of *ROCK1* and *ROCK2* in human testicular cells, with the transcriptomic data from Male Health Atlas (http://malehealthatlas.cn/). SPG: spermatogonia; SPC: spermatocytes; SPT: spermatids/sperms; SC: Sertoli cells; LC: Leydig cells; PTM: peritubular myoid cells; EC: Endothelial cells; SMC: vascular smooth muscle cells; MAC: Macrophages; MC: Mast cells; T: T cells.

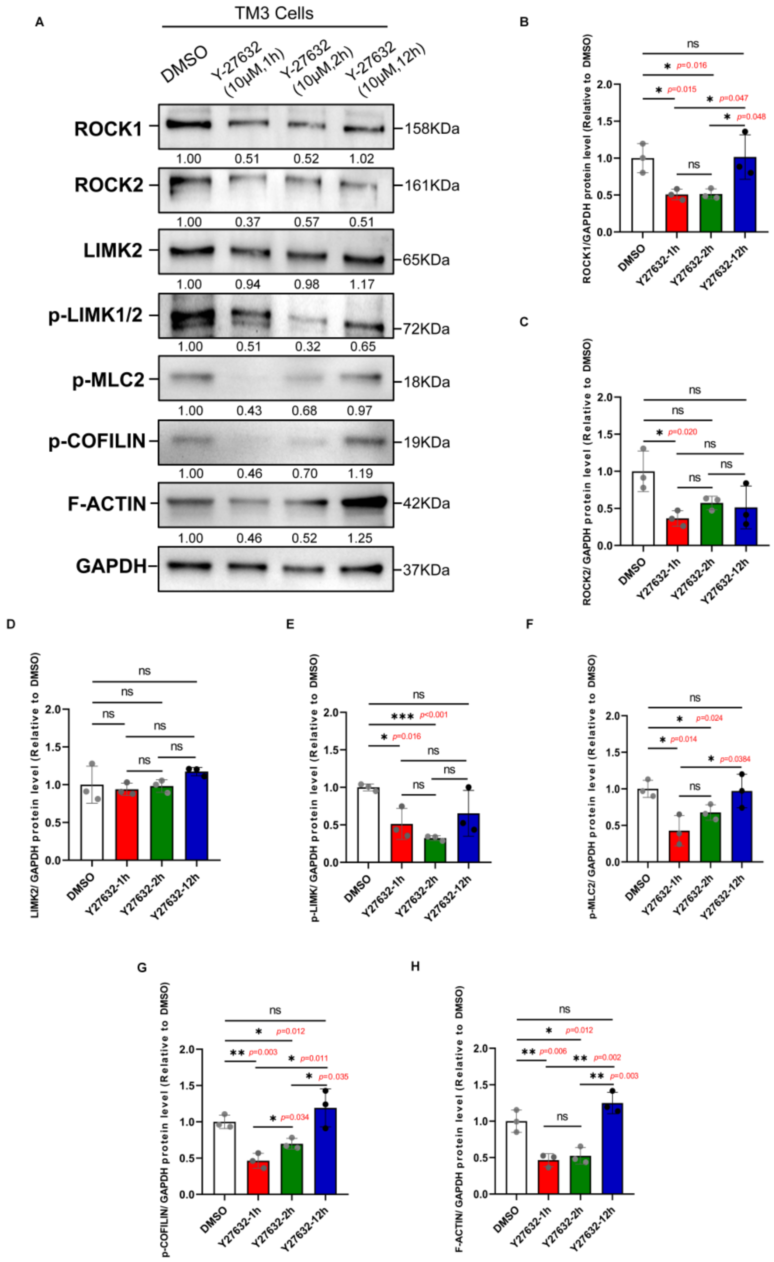

Figure S2. The time-dependent effects of ROCK inhibitor on the expression of F-actin remodeling factors in TM3 cells.

(A) The phosphorylation levels of LIMK, COFILIN, and MLC2, as well as the expression levels of F-ACTIN, were observed to change over the time course of Y27632 treatment, with GAPDH used as a loading control. (B-H) Quantification of results of Western blot analysis from (A), normalized to DMSO (n = 3). ns: not significant; *$p < 0.05$; **$p < 0.01$; ***$p < 0.001$

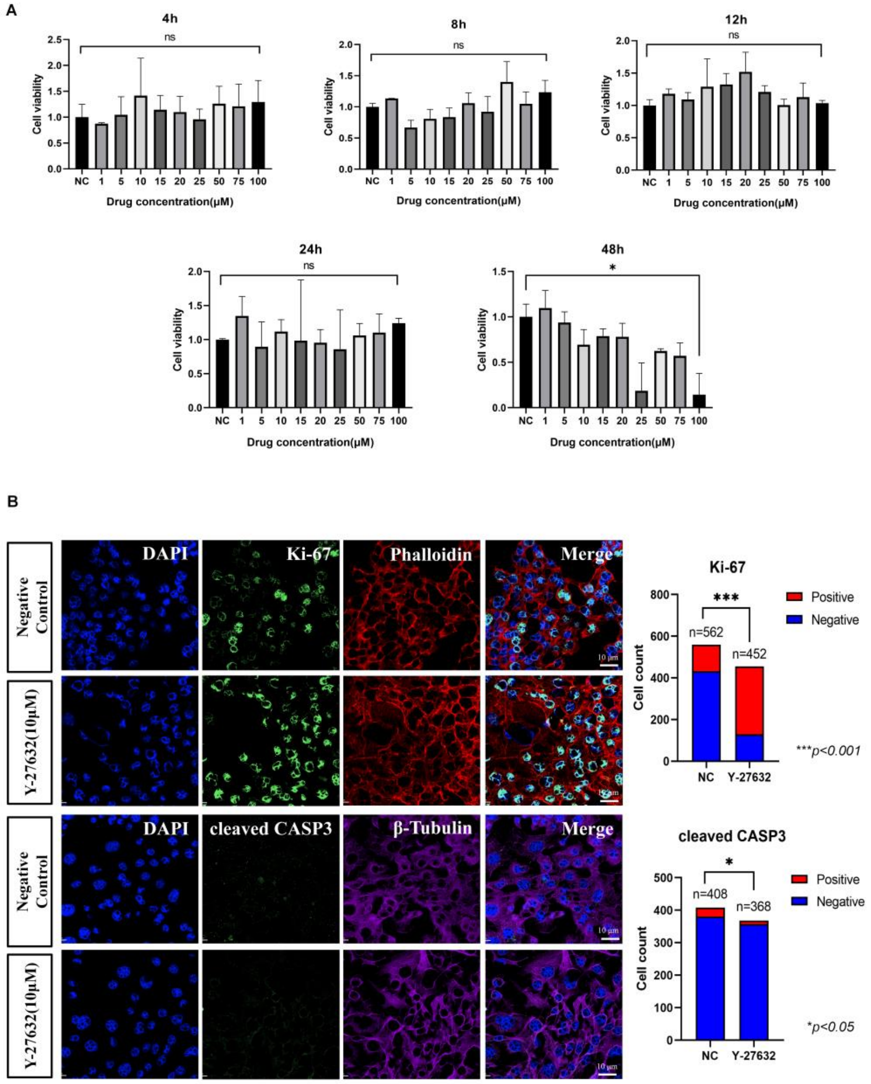

Figure S3. Y-27632 Shows No Significant Proliferative Toxicity in TM3 Cells During Its Effective Duration.
(A) The CCK-8 assay was used to evaluate the impact of Y-27632 on the proliferation and cytotoxicity of TM3 cells. Different drug concentration groups (1 μM, 5 μM, 10 μM, 15 μM, 20 μM, 25 μM, 50 μM, 75 μM, and 100 μM) and a control group (NC) were set as shown in the figure. Various drug treatment durations (4 h, 8 h, 12 h, 24 h, and 48 h) were also set as shown in the figure. "ns" indicates no significant difference; "*" indicates $p < 0.05$. (B) After 12 hours of treatment with Y-27632, the number of Ki-67 positive TM3 cells significantly increased, while the number of cleaved Caspase-3 positive cells significantly decreased. Red fluorescence indicates Phalloidin-labeled microfilaments; purple fluorescence indicates β-Tubulin-labeled microtubules; green fluorescence (top row) indicates Ki-67; green fluorescence (bottom row) indicates cleaved Caspase-3; nuclei are stained with DAPI (blue). N=3, with 10 high-power field images collected for cell counting in each experimental and control group for each repeat experiment. Scale bar: 10 μm.

A

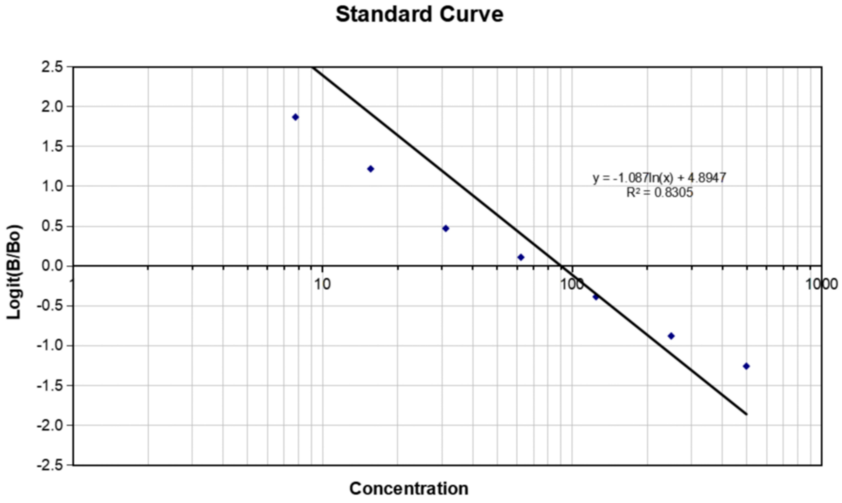

B

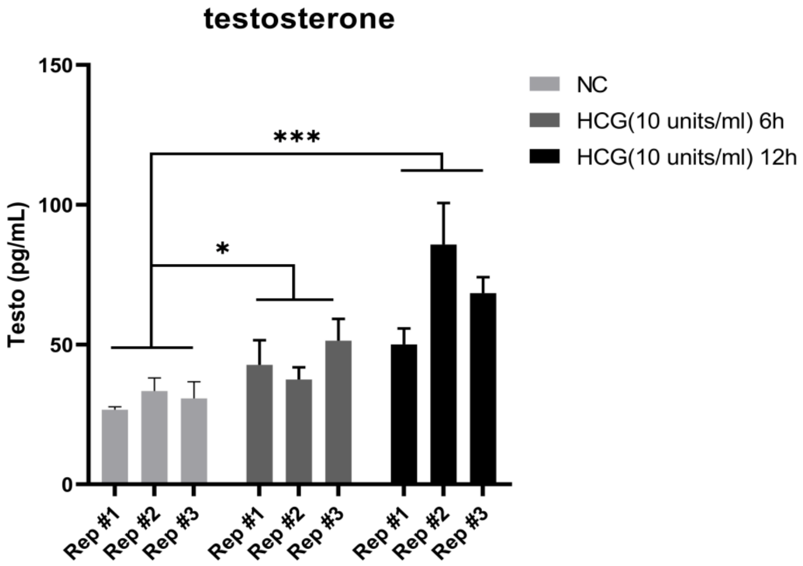

Figure S4. Detection of Testosterone in TM3 Cell Culture Medium.
(A) Standard curve for testosterone levels in cell culture medium detected by ELISA. (B) Time-dependent stimulation of testosterone synthesis and secretion in TM3 cells by hCG. Testosterone levels in the negative control group were 30.30 ± 1.950 pg/ml; in the hCG 6h group, 43.90 ± 4.050 pg/ml; and in the hCG 12h group, 68.10 ± 10.31 pg/ml. n=3, data are presented as mean ± SEM. *p<0.05; ***p<0.001.

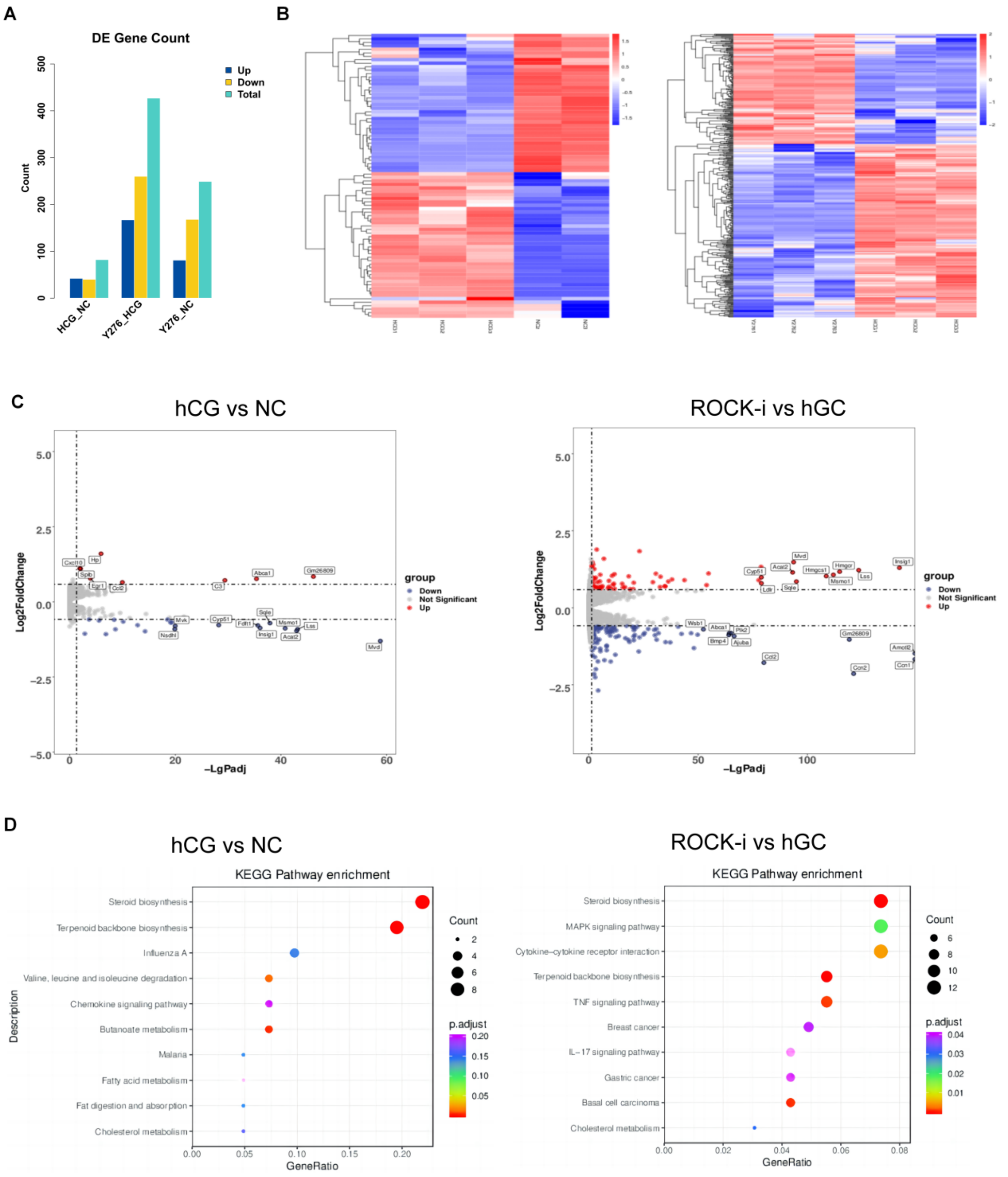

Figure S5. Differential Gene Expression and Enrichment Analysis in TM3 Cells Under hCG and ROCK-i Treatment.

(A) Differential Gene Expression Statistics. Gene expression differences were analyzed using DESeq2. The criteria for differential gene selection were: fold change ≥1.5 and *p*-value <0.05. "Up" indicates the number of genes upregulated in the treatment group compared to the control group; "Down" indicates the number of genes downregulated; "Total" indicates the total number of significantly differentially expressed genes. (B) Heatmap of Differential Gene Clustering. The heatmap shows the clustering of differentially expressed genes based on their expression levels (FPKM values). After log2 transformation, Euclidean distances were calculated and hierarchical clustering was performed. In the heatmap, rows represent genes, columns represent samples, red indicates high gene expression, and blue indicates low gene expression. The horizontal axis represents sample clustering, while the vertical axis

represents gene clustering. (C) Volcano Plot of Differential Gene Expression. The volcano plot displays the statistical significance of gene expression changes (*p*-adjust) on the horizontal axis (log10-transformed negative *p*-adjust) and the magnitude of expression change (fold change, FC) on the vertical axis (log2-transformed). Genes with *p*-adjust <0.05 are shown with threshold lines. Upregulated genes are marked in red, downregulated genes in blue, and non-significant genes in gray. The names of the top 10 most significantly upregulated and downregulated genes are displayed. (D) KEGG Enrichment Bubble Chart. The y-axis represents pathway names, the x-axis represents the ratio of genes enriched in each pathway to the total number of genes, the color indicates the *p*-adjust value (with redder colors indicating greater significance), and the bubble size represents the number of genes enriched in each pathway (with larger bubbles indicating more genes).

Table S1. Primary Antibodies Information.

Antibody	Dilution	Manufacturers	Application
rabbit anti-ROCK1 monoclonal antibody	1:1000	ab134181, Abcam	WB
rabbit anti-ROCK2 monoclonal antibody	1:10000	ab125025, Abcam	WB
rabbit anti-phospho-ROCK1 (Tyr913) polyclonal antibody	1:1000	PA5-105054, ThermoFisher Scientific	WB
rabbit anti-ROCK2 + ROCK1 monoclonal antibody	1:1000	ab45171, Abcam	WB
rabbit anti-COFILIN monoclonal antibody	1:1000	5175, Cell Signaling Technology	WB
rabbit anti-p-COFILIN monoclonal antibody	1:1000	3313, Cell Signaling Technology	WB
mouse anti-LIMK-1 monoclonal antibody	1:100	sc-515585, Santa Cruz Biotechnology	WB
mouse anti-LIMK-2 monoclonal antibody	1:100	sc-365414, Santa Cruz Biotechnology	WB
rabbit anti-phospho-LIMK1(Thr508)/LIMK2(Thr505) polyclonal antibody	1:1000	3841, Cell Signaling Technology	WB
rabbit anti-MLC2 monoclonal antibody	1:5000	ab92721, Abcam	WB
rabbit anti-phospho-MLC2 (Ser19) polyclonal antibody	1:1000	3671, Cell Signaling Technology	WB
mouse anti-F-ACTIN monoclonal antibody	1:500	ab130935, Abcam	WB
mouse anti-STAR monoclonal antibody	1:10000	67130-1-Ig, Proteintech	WB
rabbit anti-HSD3B1 monoclonal antibody	1:1000	A19266, Abclonal	WB
rabbit anti-HSD17B3 polyclonal antibody	1:2000	A7687, Abclonal	WB
rabbit anti-CYP11A1 polyclonal antibody	1:2000	13363-1-AP, Proteintech	WB
rabbit anti-CYP17A1 monoclonal antibody	1:1000	A5067, Abclonal	WB
mouse anti-SREBP2 monoclonal antibody	1:500	MAB7119, R&D Systems	WB
rabbit anti-SCAP monoclonal antibody	1:1000	ab308060, Abcam	WB
rabbit anti-phospho-SCAP (Tyr300) polyclonal antibody	1:500	SAB626, Signalway Antibody	WB
rabbit anti-INSIG1 polyclonal antibody	1:500	ab70784, Abcam	WB

rabbit anti-Lamin B1 monoclonal antibody	1:1000	13435, Cell Signaling Technology	WB
rabbit anti-β-ACTIN polyclonal antibody	1:5000	20536-1-AP, Proteintech	WB
mouse anti-GAPDH monoclonal antibody	1:10,000	60004-1-lg, Proteintech	WB
rabbit anti-ROCK1 monoclonal antibody	1:600	ab134181, Abcam	IF
rabbit anti-ROCK2 monoclonal antibody	1:200	ab125025, Abcam	IF
rabbit anti-phospho-ROCK1 (Tyr913) polyclonal antibody	1:100	PA5-105054, ThermoFisher Scientific	IF
rabbit anti-ROCK2 + ROCK1 monoclonal antibody	1:200	ab45171, Abcam	IF
mouse anti-3β-HSD monoclonal antibody	1:50	sc-515120, Santa Cruz Bio-technology	IF
rabbit anti-Vimentin monoclonal antibody	1:400	5741, Cell Signaling Tech-nology	IF
rabbit anti-Ki-67 polyclonal antibody	1:500	IHC-00395-T, Bethyl Laboratories	IF
rabbit anti-Cleaved Caspase-3 monoclonal antibody	1:800	9664, Cell Signaling Technology	IF
rabbit anti- β -ACTIN polyclonal antibody	1:1000	20536-1-AP, Proteintech	IF
rabbit anti- β -Tubulin polyclonal antibody	1:200	10094-1-AP, Proteintech	IF
mouse anti-STAR monoclonal antibody	1:600	67130-1-Ig, Proteintech	IF
mouse anti-SREBP2 monoclonal antibody	1:100	MAB7119, R&D Systems	IF
rabbit anti-phospho-MLC2 (Ser19) polyclonal antibody	1:100	3671, Cell Signaling Technology	IF
rabbit anti-phospho-SCAP (Tyr300) polyclonal antibody	1:100	SAB626, Signalway Antibody	IF

Table S2. qPCR Primer Sequences.

Gene	Forward Primer (5'->3')	Reverse Primer (5'->3')
Gapdh	GAGAGTGTTTCCTCGTCCCG	ACTGTGCCGTTGAATTTGCC
Abca1	AAAACCGCAGACATCCTTCAG	CATACCGAAACTCGTTCACCC
ApoE	GCTGGGTGCAGACGCTTT	TGCCGTCAGTTCTTGTGTGACT
Osbp	AGGAGGAAACAGTGAAGGCTGCAAC	CAATCACCTGGAGAGAGCCTTCCGG
Orp4	GAGACCTTCGAGCTGGACCGTATGG	CTCACCACTCCAGTCACCTTTCGGG
Orp8	TCCGAGCTACTTCAGAGTCAGATGG	TTTAAGGGCTCCACAGGCTCAGGGT
Orp9	GCCGAATGATACTGAAGAGAACGCAG	AAGGCTGCGGGACTCATACTCATTCT
Orp10	CAGAAGAGGAACACAACTCACAGCC	AGGAGATGGGCGGATGATGGGATAC
Hmgcs	GCCGTGAACTGGGTCGAA	GCATATATAGCAATGTCTCCTGCAA
Hmgcr	CTTGTGGAATGCCTTGTGATTG	AGCCGAAGCAGCACATGAT
Ldlr	AGGCTGTGGGCTCCATAGG	TGCGGTCCAGGGTCATCT
Mvk	GGACACGAGCTTTCTTGAGC	GCAGATTGCCAGGTACAGGT
Mvd	AAGCAGACGGGCAGTACAGT	CCTGGAGGTGTCATTGAGGT
Lss	GCTGCATGTGGTGTATGGAC	GAGAAACGTGCTCCTGGAAG
Fdft1	TCCCTGACGTCCTCACCTAC	GGGGATCCGGTGATAAATCT
Cyp51	CACACATTGCCACAGGGAGA	GAAGTGGCCCAACTACACGA
Insig1	TCACAGTGACTGAGCTTCAGCA	TCATCTTCATCACACCCAGGAC
Rock1	GACTGGGGACAGTTTTGAGAC	GGGCATCCAATCCATCCAGC
Rock2	TTGGTTCGTCATAAGGCATCAC	TGTTGGCAAAGGCCATAATATCT
Msmo1	AAGGTTTCGGGAACTGGAGG	AAGGCAACGTCAACTTCAGC
Sqle	CGCAGCGGTTACTCTGGTTA	ATTCCTCCTCAAGCAAGCCC
Scap	ATTTGCTCACCGTGGAGATGTT	GAAGTCATCCAGGCCACTACTAATG
Abcg8	CTGTGGAATGGGACTGTACTTC	GTTGGACTGACCACTGTAGGT
Acat1	CAGGAAGTAAGATGCCTGGAAC	TTCACCCCCTTGGATGACATT
Acat2	CCCGTGGTCATCGTCTCAG	GGACAGGGCACCATTGAAGG
Abcg1	CTTTCCTACTCTGTACCCGAGG	CGGGGCATTCCATTGATAAGG
